# CircDYM ameliorates depressive-like behavior by targeting miR-9 to regulate microglial activation via HSP90 ubiquitination

**DOI:** 10.1038/s41380-018-0285-0

**Published:** 2018-11-09

**Authors:** Yuan Zhang, Longfei Du, Ying Bai, Bing Han, Cancan He, Liang Gong, Rongrong Huang, Ling Shen, Jie Chao, Pei Liu, Hongxing Zhang, Haisan Zhang, Ling Gu, Junxu Li, Gang Hu, Chunming Xie, Zhijun Zhang, Honghong Yao

**Affiliations:** 1 Department of Pharmacology, School of Medicine, Southeast University, Nanjing, 210009 Jiangsu China; 2 Department of Neurology of Affiliated ZhongDa Hospital, Institute of Neuropsychiatry of Southeast University, Nanjing, 210009 Jiangsu China; 3 Department of Physiology, School of Medicine, Southeast University, Nanjing, 210009 Jiangsu China; 4 Department of Epidemiology and Biostatistics, School of Public Health, Southeast University, Nanjing, 210009 Jiangsu China; 5 Department of Psychology of Xinxiang Medical University, Xinxiang, Henan 453003 China; 6 Second Affiliated Hospital of Xinxiang Medical University, Xinxiang, Henan 453003 China; 7 Basic Medical College, Nanjing University of Chinese Medicine, Nanjing, 210046 Jiangsu China; 8 Department of Pharmacology and Toxicology, University at Buffalo, Buffalo, 14203 NY USA; 9 Jiangsu Key Laboratory of Neurodegeneration, Department of Pharmacology, Nanjing Medical University, Nanjing, 210029 Jiangsu China; 10 Institute of Life Sciences, Key Laboratory of Developmental Genes and Human Disease, Southeast University, Nanjing, 210096 Jiangsu China

**Keywords:** Neuroscience, Molecular biology

## Abstract

Circular RNAs (circRNAs), highly expressed in the central nervous system, are involved in various regulatory processes and implicated in some pathophysiology. However, the potential role of circRNAs in psychiatric diseases, particularly major depressive disorder (MDD), remains largely unknown. Here, we demonstrated that circular RNA DYM (circDYM) levels were significantly decreased both in the peripheral blood of patients with MDD and in the two depressive-like mouse models: the chronic unpredictable stress (CUS) and lipopolysaccharide (LPS) models. Restoration of circDYM expression significantly attenuated depressive-like behavior and inhibited microglial activation induced by CUS or LPS treatment. Further examination indicated that circDYM functions as an endogenous microRNA-9 (miR-9) sponge to inhibit miR-9 activity, which results in a downstream increase of target-HECT domain E3 ubiquitin protein ligase 1 (HECTD1) expression, an increase of HSP90 ubiquitination, and a consequent decrease of microglial activation. Taken together, the results of our study demonstrate the involvement of circDYM and its coupling mechanism in depression, providing translational evidence that circDYM may be a novel therapeutic target for depression.

## Introduction

Major depressive disorder (MDD), characterized by emotional dysfunction, is one of the most prevalent psychiatric disorders worldwide and a major public health concern associated with grave consequences [1–4]. MDD arises from a combination of genetic and environmental factors [5] with stress being a major risk factor that can lead to the precipitation of depression [6]. With a lack of clinical biomarkers, MDD is currently diagnosed by subjective symptoms. Due to the heterogeneity of depression and low recognition rate of high-risk individuals, symptom-based diagnosis is clinically inadequate and does not lead to accurate classification of depression [7, 8]. Furthermore, there is a lack of effective antidepressant drugs; roughly one-third of patients experience an inadequate response [9, 10]. Further understanding of the detailed mechanisms of depression is needed to identify more effective therapeutic targets.

Circular RNAs (circRNAs), generated by joining the 3’ and 5’ splice sites in the primary linear transcript, have recently been identified as a naturally occurring non-coding RNA family that is highly represented in the eukaryotic transcriptome [11]. These endogenous RNAs are characterized by a stable loop structure, evolutionary conservation between different species, and high tissue/developmental-stage-specific expression [12]. While the majority of circRNAs generated from exons are distributed in the cytoplasm with a post-transcriptional regulation function [13–15], a class of intron-containing exonic and intronic circRNAs are found predominantly in the nucleus where they promote the transcription of parental genes [16]. CircRNAs have been shown to be involved in a range of physiological processes and disease conditions, including neural development and plasticity; cell growth; Alzheimer’s disease; and heart senescence, hypertrophy, and failure [17–21]. Previous study investigated the association of circRNAs with depression in type 2 diabetes mellitus (T2DM) and found 183 circRNAs significantly upregulated and 64 circRNAs significantly downregulated in the depression group compared to patients without depression. Four of these circRNAs were predicted to target miRNA and genes related to depression, suggesting that there may be a relationship between circRNAs and the pathogenesis of depression in T2DM patients [22]. Only one study in MDD showed that altered expression of has_circRNA_103636 in peripheral blood mononuclear cells may be a potential diagnostic biomarker for MDD [23]. However, little is known regarding the biological function of circRNAs in the pathogenesis of MDD.

Circular RNA DYM (circDYM), derived from exons 4, 5, and 6 of the DYM gene, acts to be a microRNA-9 (miR-9) sponge. Previous studies have indicated that the expression of miR-9 was increased in the prefrontal cortex of a chronic unpredictable stress (CUS) model [24, 25]. In our previous work, we demonstrated that silencing miR-9 inhibited the hippocampal microglial activation induced by lipopolysaccharide (LPS) [26]. Microglia play an important role in immune surveillance and are known to be actively involved in various neurologic pathologies [27, 28]. Microglial activation with subsequent inflammatory cytokine release also mediates the effects of stress [29–31]; these processes have been implicated as a major trigger of depression in both human and animal models [32–34]. At different stages of depression, the functional status of microglia is diverse, and they exert different regulatory effects on neurons in different functional states [30, 31, 35].

Together, these findings suggest that circDYM/miR-9 may be involved in MDD via the regulation of microglial activation. In this study, we investigate this hypothesis and its mechanism.

## Materials and methods

### Study approval and human subjects

The ethics committee at Henan Provincial Mental Hospital, Affiliated to Xinxiang Medical University approved this research protocol (approval ID: 2017-08), and all participants or their legally authorized representatives provided written informed consent to participate in the study. MDD patients were recruited through inpatients at the Department of Psychiatry in Henan Provincial Mental Hospital. Healthy control subjects were enrolled through local community posting and media advertising. Inclusion and Exclusion Criteria and Behavior Measurements were described in the Supplementary Information.

### Animals

Adult male C57BL/6J mice (25.0–30.0 g, 6–8 weeks old) were purchased from the Model Animal Research Center of Nanjing University (Nanjing, China) and randomly assigned to experimental groups. All animals were housed under a constant temperature and humidity and a 12-h light/12-h dark cycle with the lights on at 7:00 AM. Food and water were available *ad libitum*. All animal procedures were performed in strict accordance with the Animal Research: Reporting of *In Vivo* Experiments Guidelines. The care and use of animals were reviewed and approved by the Institutional Animal Care and Use Committee at the Medical School of Southeast University.

### Microinjection of circDYM overexpression lentivirus

All mice were weighed before experiments and randomly assigned to different groups. C57BL/6J male mice were microinjected bilaterally with either the circControl-GFP lentivirus or circDYM-GFP lentivirus (1 μl of 1 × 10^9^ viral genomes/µl, Hanbio, Shanghai, China) into the hippocampus using the following microinjection coordinates: 2.06 mm behind the bregma and ± 1.5 mm lateral from the sagittal midline at a depth of 2 mm from skull surface. To evaluate the effect of circDYM overexpression on LPS-induced microglia activation, one week after lentivirus microinjection, we intraperitoneally injected mice with LPS (1 mg/kg) or saline for 5 successive days. Mice were randomly divided into four groups: circControl + saline (*n* = 11); circDYM + saline (*n* = 11); circControl + LPS (*n* = 11); and circDYM + LPS (*n* = 11). To evaluate the effect of circDYM overexpression on CUS-induced microglia activation, 1 week after lentivirus microinjection, we exposed mice to a CUS or control protocol for 5 weeks. Mice were divided into four groups: circControl + Control (*n* = 11); circDYM + Control (*n* = 11); circControl + CUS (*n* = 11); and circDYM + CUS (*n* = 11).

### CUS protocol

To induce chronic stress in mice, we used a previously validated CUS protocol with some modifications [36]. Mice were exposed to various, randomly scheduled, low-intensity social and environmental stressors 2–3 times a day for 5 weeks. The stressors included the following (1) food deprivation for 24 h, (2) water deprivation for 24 h, (3) overnight illumination, (4) absence of sawdust in cage for 24 h, (5) moistened sawdust with water for 24 h, (6) forced swimming at 8 °C for 5 min, (7) tail nipping (1 cm from the tip of the tail), (8) physical restraint for 6 h, and (9) 45° cage-tilt along the vertical axis for 3 h.

### Behavioral tests

Behavioral tests were conducted after CUS or LPS treatment. All tests were carried out between 9:00 and 17:00 h in a sound-attenuated room under low-intensity light and were scored by the same rater. Mice were habituated in the room for at least 3 h before the tests. Behavior was monitored through a video camera positioned in front of the testing apparatuses, and the images were later analyzed with a Plexon research solutions system (Plexon Inc, Dallas, TX, USA) by an experienced researcher who was blind to the treatment option of the animals tested. Animals completed the sucrose preference test (SPT), tail suspension test (TST), and forced swim test (FST) as described in the Supplementary Information.

### Immunostaining and image analysis

As described in our previous study [26], the immunostaining was performed as described in the Supplementary Information. Images were captured using a microscope (Zeiss, Oberkochen, Germany, ImagerM2). Computer-based cell tracing software Neurolucida 360 (MBF Bioscience, Williston, VT, USA) was used for three-dimensional (3D) reconstruction of Iba-1-positive cells within the hippocampus. NeuroExplorer (MBF Bioscience, Williston, VT, USA) was used to analyze 10 cells per animal. Sholl analysis was used to determine branch tree morphology by placing three-dimensional concentric circles in 5 mm increments starting at 5 mm from the soma.

### Transient transfection with clustered regularly interspaced short palindromic repeats (CRISPR)/CRISPR associated protein 9 (Cas9)

Primary mouse microglia and BV-2 cells were transiently transfected with CRISPR/CRISPR Cas9 plasmids according to the manufacturer’s recommended protocol (Santa Cruz^®^, California, USA) to delete/upregulate HECT domain E3 ubiquitin protein ligase 1 (HECTD1) and examine the downstream effects. The transfection efficiency was determined via western blotting. In brief, cells were seeded at 2 × 10^5^ cells per well in a 6-well plate in 3 ml of antibiotic-free standard growth medium and grown to 40–80% confluency. Then, 300 µl of the Plasmid DNA/UltraCruz^®^ Transfection Reagent Complex, consisting of 2 µg plasmid DNA and 10 µl UltraCruz^®^ Transfection Reagent in Plasmid Transfection Medium, was added dropwise to each well. Thereafter, gentle mixing was performed by swirling the plate, and the cells were incubated and cultured for 24–72 h under normal conditions prior to subsequent experiments.

### Transient transfection with synthetic miR-9-5p mimic and inhibitor

miR-9-5p mimic control (miR-Con), miR-9-5p mimic (miR-9), miR-9-5p inhibitor control (anti-miR-Con), and miR-9-5p inhibitor (anti-miR-9) were synthesized by Ribobio (Guangzhou, China). Primary mouse microglia and BV-2 cells were seeded at 1 × 10^5^ cells per well in a 24-well plate in 1 ml of antibiotic-free standard growth medium and grown to 40–80% confluency. Then, cells were transfected with 50 nM miR-Con or miR-9 or with 100 nM anti-miR-Con or anti-miR-9. 1 μl RiboFECT™CP reagent (RiboBio, Guangzhou, China) was added to each well according to the manufacturer’s protocols. The medium containing RiboFECT™CP reagent was replaced after 24 h of transfection. Transfection efficiency was determined via western blotting.

### Affinity isolation assay with biotinylated miRNA

According to a previously described protocol [14, 37], bound RNAs were purified using TRIzol to measure circDYM and GAPDH levels. The 3′-biotinylated WT miR-9 (Bio-miR-9-WT) sequence was 5′-UCUUUGGUUAUCUAGCUGUAUGA-3′, and the 3’-biotinylated mutant miR-9 (Bio-miR-9-mut) sequence was 5′-UGGGCCCUUAUCUAGCUGUAUGA-3′. All 3’-biotinylated miRNAs were synthesized by Shanghai GenePharma (Shanghai, China).

### Affinity isolation assay with biotinylated DNA probes

According to a previously described protocol [37], the sequence of the 3’-biotinylated circDYM probe was 5′-AAACGAGGATTGTTTTCAAAAGAGTGGAATATCAG-3′, and the sequence of the 3′-biotinylated random probe was 5′-AAACAGTACTGGTGTGTAGTACGAGCTGAAGCTAC-3′. All probes were synthesized by Invitrogen (Shanghai, China).

### Western blotting (WB) and other experiments

WB was performed as previously described [38]. Real-time polymerase chain reaction (PCR), fluorescence in situ hybridization (FISH), FISH in combination with immunostaining, cell cultures, transduction of microglia with lentiviruses, luciferase activity assays, and enzyme-linked immunosorbent assay (ELISA) were performed as described in the Supplementary Information. Detailed information about the primers and antibodies was provided in the Supplementary Table 1 and Table 2, respectively.

### Statistics

The individual statistical analyses used for the different experiments are described in the respective figure legends. Power analysis software (Hintze, J. NCSS, LLC. Kaysville, Utah, USA. www.ncss.com) was used to calculate the sample power for the clinical study. Sample size required for animal study was empirically based upon the results of previous experiments and similar to that generally used in the field. Shapiro-Wilkes tests were used to assess normality in the distribution for each group; only miR-9 in blood sample was found to be non-Gaussian and was analyzed using non-parametric test. F-tests were used for homogeneity of variance and all statistically compared groups are with similar variance (*p* > 0.05). Correlation was measured using Pearson’s correlation coefficient and the interactive effect was analyzed using Multivariate Linear Regression. Statistical analysis between two groups was tested using Student’s *t* test or Mann–Whitney *U* test, where appropriate. The comparisons among groups were tested by two-way analysis of variance (ANOVA) followed by Bonferroni’s post hoc multiple comparison tests. All the data are presented as means ± SEM. Results were judged to be statistically significant if *p* < 0.05. Statistical analysis was performed using SPSS 22.0 software (IBM, USA).

## Results

### Downregulation of circDYM in the MDD patients and the depressive-like animal models

To determine whether miR-9 is involved in depression, we examined miR-9 levels in normal subjects and MDD patients. The sociodemographic and clinical characteristics of these subjects are listed in Supplementary Tables 3–5. As shown in Supplementary Figure 1A, miR-9 levels were significantly increased in MDD patients compared with those of age- and gender-matched normal controls (*p* = 0.0018). Furthermore, we observed a positive correlation between miR-9 levels and the scores of Hamilton Anxiety Scale (HAMA) (Pearson correlation coefficient *r* = 0.341, *p* = 0.023) (Supplementary Figure 1B). Further linear regression analysis revealed that MDD patients with higher miR-9 expression and lower childhood trauma questionnaire (CTQ) scores showed more severe depressive symptoms (Supplementary Figure 1C). The analysis of receiver-operating characteristic (ROC) curve revealed that miR-9 had a significant area under the curve (AUC) of 0.757 with 0.639 sensitivity and 0.9 specificity (Supplementary Figure 1D).

**Fig. 1:**
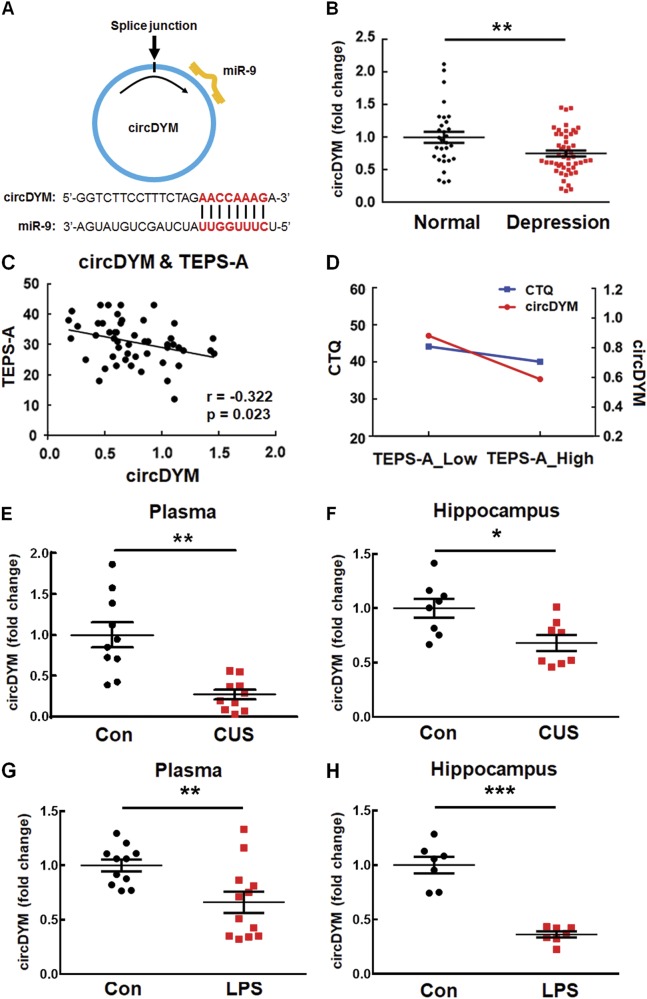
CircDYM was downregulated in the MDD patients and the depressive-like animal models. **a** circDYM contains one site that is complementary to miR-9 according to the bioinformatics program RNAhybrid. **b** Levels of circDYM were decreased in the peripheral blood of MDD patients (*n* = 50) compared with those of normal controls (*n* = 30). All the data were presented as mean ± SEM. **p < 0.01 versus normal control group using Student’s *t* test. **c** Correlation between circDYM expression and TEPS-A scores using the Pearson’s correlation coefficient. **d** Interactive effects of circDYM and CTQ scores on TEPS-A scores in MDD patients using Multivariate Linear Regression. MDD patients with lower circDYM and CTQ scores showed more severe depressive symptoms. **e** Expression of circDYM in the plasma of CUS mice. After exposure to CUS for 5 weeks, mice were sacrificed. Plasma was collected, and RNA was isolated for examination of circDYM by real-time PCR. All data were presented as mean ± SEM. *n* = 10 mice/group, **p < 0.01 versus Control using Student’s *t* test. **f** Expression of circDYM in the hippocampus of CUS mice. After exposure to CUS for 5 weeks, mice were sacrificed. The hippocampus was collected, and RNA was isolated for examination of circDYM by real-time PCR. All data were presented as mean ± SEM. *n* = 8 mice/group, *p < 0.05 versus Control using Student’s *t* test. **g** Expression of circDYM in the plasma of mice treated with LPS. Mice were intraperitoneally injected with LPS (1 mg/kg) or saline for 5 successive days and then sacrificed. Plasma was collected, and RNA was isolated for examination of circDYM by real-time PCR. All data were presented as mean ± SEM. *n* = 11-12 mice/group, **p < 0.01 vs. Control using Student’s *t* test. **h** Expression of circDYM in the hippocampus of mice treated with LPS. Mice were intraperitoneally injected with LPS (1 mg/kg) or saline for 5 successive days and then sacrificed. The hippocampus was collected, and RNA was isolated for examination of circDYM by real-time PCR. All the data were presented as mean ± SEM. *n* = 7 mice/group, ***p < 0.001 vs. Control using Student’s *t* test

Since circDYM contains one miR-9 target site (Fig. 1a), we next sought to examine the expression of circDYM in the MDD patients by employing divergent primers specific to circDYM (Supplementary Figure 2). We found that circDYM levels in MDD subjects were significantly decreased compared with those in healthy controls (*p* = 0.0062) (Fig. 1b). Furthermore, we observed a negative correlation between circDYM levels and the scores of Temporal Experience of Pleasure Scale-Anticipatory Pleasure (TEPS-A) (Pearson correlation coefficient *r* = -0.322, *p* = 0.023) (Fig. 1c). Further linear regression analysis revealed that MDD patients with lower expression of circDYM and lower CTQ scores showed more severe depressive symptoms (Fig. 1d).

Consistent with these clinical findings, circDYM expression levels decreased in the plasma and hippocampus of CUS (Fig. 1e, f) and LPS (Fig. 1g, h) mice compared with those in the control groups. Using real-time PCR, we verified the expression of circDYM in organs other than the brain, such as the heart, liver, spleen, lung, and kidney. As shown in Supplementary Figure 3, circDYM was highly expressed in the brain compared to other tissues.

### Overexpression of circDYM ameliorated depressive-like behavior induced by CUS

**Fig. 2:**
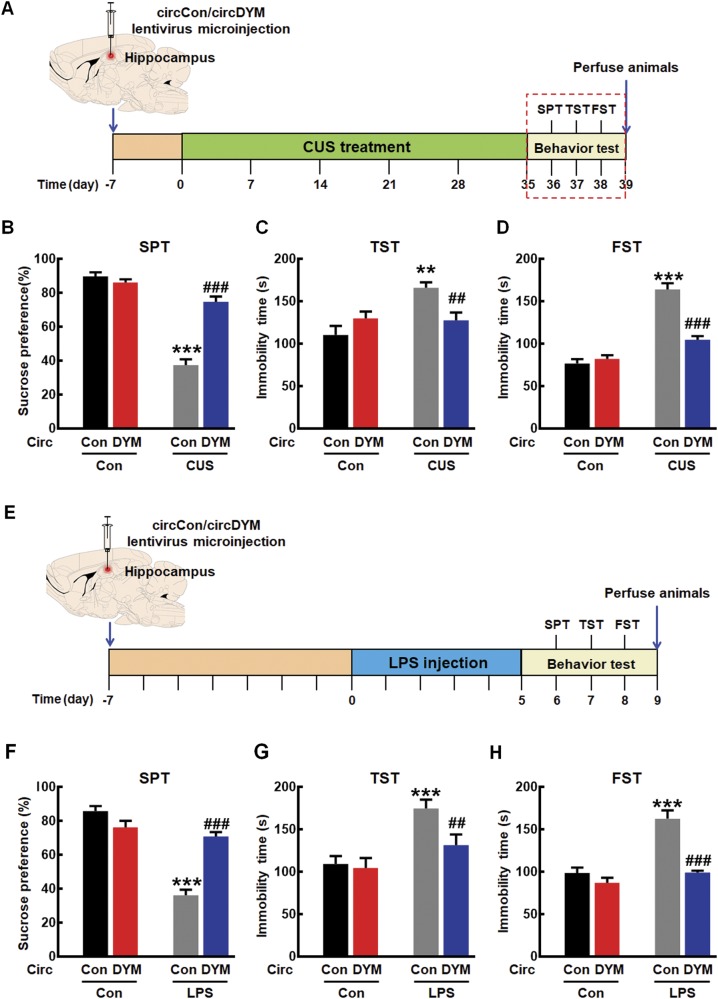
Overexpression of circDYM ameliorated depressive-like behavior. **a** Timeline of experimental procedure in the CUS-induced mouse depressive model. **b**–**d** Effects of circDYM-GFP lentivirus microinjection on the depressive-like behavior in CUS mice. One week after circControl/circDYM-GFP lentivirus microinjection, mice were exposed to a CUS or control protocol (*n* = 11 mice/group). SPT (**b**), TST (**c**), and FST (**d**) were measured after 5 weeks of CUS exposure. All data were presented as mean ± SEM. (SPT, circDYM: F_(1,40)_ = 33.454, *P* < 0.001; CUS: F_(1,40)_ = 119.263, *P* < 0.001; interaction: F_(1,40)_ = 49.660, *P* < 0.001. TST, circDYM: F_(1,40)_ = 4.217, *P* < 0.05; CUS: F_(1,40)_ = 7.676, *P* < 0.01; interaction: F_(1,40)_ = 4.795, *P* < 0.05. FST, circDYM: F_(1,40)_ = 22.168, *P* < 0.001; CUS: F_(1,40)_ = 91.678, *P* < 0.001; interaction: F_(1,40)_ = 31.976, *P* < 0.001). **e** Timeline of experimental procedure in the LPS-induced mouse depressive model. **f**–**h** Effects of circDYM-GFP lentivirus microinjection on the depressive-like behavior in LPS mice. One week after circControl/circDYM-GFP lentivirus microinjection, mice were intraperitoneally injected with LPS (1 mg/kg, *n* = 11 mice/group) or saline (*n* = 11 mice/group) for 5 successive days. SPT (**f**), TST (**g**), and FST (**h**) were then measured. All data were presented as mean ± SEM. (SPT, circDYM: F_(1,40)_ = 14.492, *P* < 0.001; LPS: F_(1,40)_ = 69.880, *P* < 0.001; interaction: F_(1,40)_ = 45.436, *P* < 0.001. TST, circDYM: F_(1,40)_ = 6.148, *P* < 0.05; LPS: F_(1,40)_ = 16.830, *P* < 0.001; interaction: F_(1,40)_ = 4.140, *P* < 0.05. FST, circDYM: F_(1,40)_ = 32.290, *P* < 0.001; LPS: F_(1,40)_ = 32.757, *P* < 0.001; interaction: F_(1,40)_ = 15.301, *P* < 0.001). **P < 0.01 and ***p < 0.001 vs. circControl Control group; ^##^*p* < 0.01 and ^###^*p* < 0.001 vs. circControl treated with CUS/LPS group. SPT sucrose preference test, TST tail suspension test, FST forced swim test

Having determined that circDYM was decreased in CUS and LPS mice, we next sought to verify the role of circDYM in vivo by microinjecting either the circControl-GFP lentivirus or circDYM-GFP lentivirus into the hippocampus of C57BL/6J mice. The mice were monitored to determine the role of circDYM in depression pathogenesis, as illustrated in Fig. 2a. After the lentivirus microinjection, GFP was widely expressed in the hippocampus, and a certain number of Iba-1-positive cells co-localized with GFP (Supplementary Figure 4A). We then examined the efficacy of circControl/circDYM-GFP lentivirus transduction in vivo. As shown in Supplementary Figure 4B, increased expression of circDYM was observed in circDYM-injected mice compared with circControl-injected mice. One week after lentivirus microinjection, mice were exposed to a CUS protocol for 5 weeks followed by depressive-like behavior tests. As shown in Fig. 2b, the CUS treatment decreased mice’s sucrose preference compared with that of the control group. This deficit was significantly ameliorated by circDYM overexpression. Two other behavioral tests (TST and FST) were employed to evaluate the effect of circDYM on the depressant activity. In both TST (Fig. 2c) and FST (Fig. 2d), immobility time was significantly higher in CUS mice than in control mice, which was significantly ameliorated in circDYM-injected mice. As shown in Fig. 2e, 1 week after lentivirus microinjections, mice were injected with LPS for 5 days followed by behavioral tests. Findings from the LPS model were consistent with those from the CUS model (Fig. 2f-h).

### Restoration of circDYM expression inhibited microglial activation in vivo

**Fig. 3:**
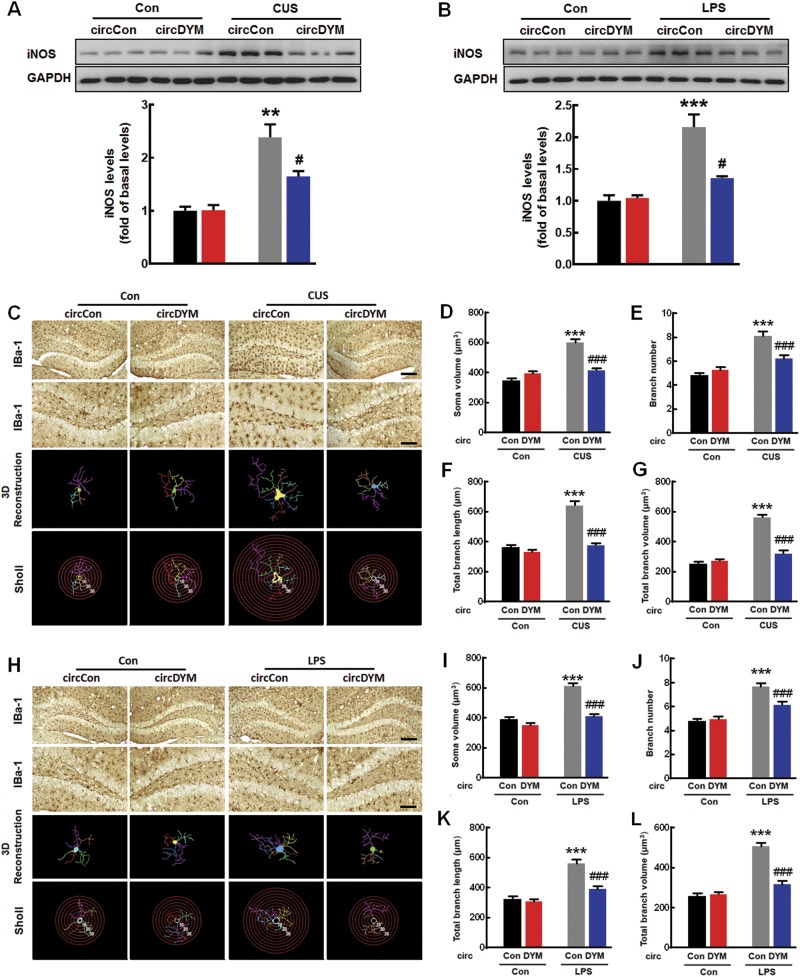
Overexpression of circDYM inhibited microglial activation in vivo. **a**, **b** Effect of circDYM overexpression on iNOS levels in CUS (**a**) and LPS (**b**) models. Mice were microinjected with the circControl/circDYM-GFP lentivirus in the hippocampus. One week after microinjection, mice were exposed to a CUS protocol for 5 weeks, or they were intraperitoneally injected with LPS (1 mg/kg) for 5 successive days. Three representative immunoblots were presented from 6 mice/group. All data were presented as mean ± SEM. (CUS model, circDYM: F_(1,20)_ = 10.261, *P* < 0.01; CUS: F_(1,20)_ = 55.047, *P* < 0.001; interaction: F_(1,20)_ = 10.918, *P* < 0.01. LPS model, circDYM: F_(1,20)_ = 11.488, *P* < 0.01; CUS: F_(1,20)_ = 43.685, *P* < 0.001; interaction: F_(1,20)_ = 14.374, *P* < 0.01). **c**–**g** Effect of circDYM on microglial activation induced by CUS. Representative images of microglial immunostaining for Iba-1 in mice hippocampus, followed by 3D reconstruction and Sholl analysis (**c**). Scale bars: 200 μm (upper panel) and 100 μm (lower panel). Average soma size (**d**), branch number (**e**), total branch length (**f**), and total branch volume (**g**). All data were presented as mean ± SEM. *n* = 5 mice/group, 50 cells/group. (Average soma size, circDYM: F_(1,196)_ = 15.035, *P* < 0.001; CUS: F_(1,196)_ = 60.454, *P* < 0.001; interaction: F_(1,196)_ = 42.132, *P* < 0.001. Branch number, circDYM: F_(1,196)_ = 6.649, *P* < 0.05; CUS: F_(1,196)_ = 57.645, *P* < 0.001; interaction: F_(1,196)_ = 17.259, *P* < 0.001. Total branch length, circDYM: F_(1,196)_ = 60.587, *P* < 0.001; CUS: F_(1,196)_ = 73.005, *P* < 0.001; interaction: F_(1,196)_ = 38.486, *P* < 0.001. Total branch volume, circDYM: F_(1,196)_ = 47.866, *P* < 0.001; CUS: F_(1,196)_ = 127.414, *P* < 0.001; interaction: F_(1,196)_ = 67.190, *P* < 0.001). **h**–**l** Effect of circDYM on microglial activation induced by LPS. Representative images of microglial immunostaining for Iba-1 in mice hippocampus, followed by 3D reconstruction and Sholl analysis (**h**). Scale bars: 200μm (upper panel) and 100 μm (lower panel). Average soma size (**i**), branch number (**j**), total branch length (**k**), and total branch volume (**l**). All the data were presented as mean ± SEM. *n* = 5 mice/group, 50 cells/group. (Average soma size, circDYM: F_(1,196)_ = 50.359, *P* < 0.001; LPS: F_(1,196)_ = 69.865, *P* < 0.001; interaction: F_(1,196)_ = 23.044, *P* < 0.001. Branch number, circDYM: F_(1,196)_ = 7.061, *P* < 0.01; LPS: F_(1,196)_ = 63.546, *P* < 0.001; interaction: F_(1,196)_ = 10.267, *P* < 0.01. Total branch length, circDYM: F_(1,196)_ = 22.037, *P* < 0.001; LPS: F_(1,196)_ = 66.660, *P* < 0.001; interaction: F_(1,196)_ = 15.319, *P* < 0.001. Total branch volume, circDYM: F_(1,196)_ = 36.990, *P* < 0.001; LPS: F_(1,196)_ = 97.182, *P* < 0.001; interaction: F_(1,196)_ = 45.217, *P* < 0.001). **p < 0.01 and ***p < 0.001 versus circControl Control group; ^#^*p* < 0.05 and ^###^*p* < 0.001 versus circControl treated with CUS/LPS group

Since circDYM ameliorated depressive-like behavior induced by CUS and LPS, we next examined the mechanisms underlying this process. Mice that received circDYM lentivirus microinjection and then treated with CUS or LPS were examined for neuroinflammation as determined by iNOS expression as well as cytokine level. As shown in Fig. 3a, overexpression of circDYM significantly inhibited the CUS-induced increase in iNOS expression and cytokines (IL-6, IL-1β, MCP-1, and TNF-α) (Supplementary Figure 5) in the hippocampus. This finding was confirmed in LPS-injected mice (Fig. 3b, Supplementary Figure 6).

Given that microglia play an important role in neuroinflammation and that circDYM was expressed in microglia (Supplementary Figure 7), we further examined the effect of circDYM on microglial activation and morphology in the hippocampus using Iba-1 staining. CUS exposure induced microglial activation compared with the control protocol, as indicated by CUS mice’s increased soma size (Fig. 3c, d) and hyper-ramification of microglia characterized by the significantly increased branch number, length, and volume (Fig. 3e–g). These effects were significantly attenuated by circDYM microinjection. The same results were found in the LPS model (Fig. 3h–l).

### Restoration of circDYM expression inhibited microglial activation by targeting miR-9 in vitro

**Fig. 4:**
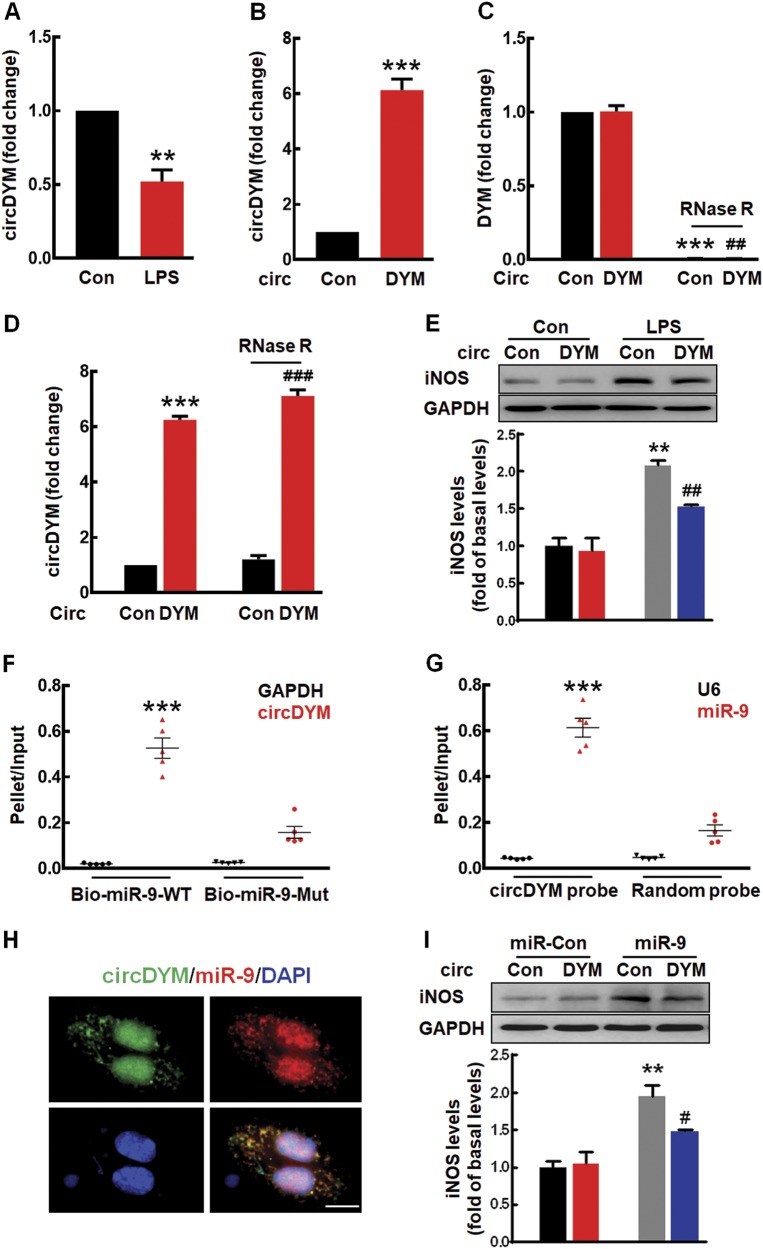
CircDYM overexpression inhibited microglial activation by targeting miR-9 in vitro. **a** Effect of LPS on the expression of circDYM in primary mouse microglia. **b** Expression of circDYM in primary mouse microglia transduced with the circControl/circDYM-GFP lentivirus. All the data were presented as mean ± SEM of 3 independent experiments. **p < 0.01 and ***p < 0.001 vs. Control using Student’s *t* test. **c**, **d** The levels of DYM mRNA (**c**) and circDYM (**d**) in BV-2 cells. Cells were transduced with the circControl/circDYM-GFP lentivirus and incubated with or without RNase R. Total RNA extracted from BV-2 cells and the level of DYM mRNA or circDYM were detected by real-time PCR. All data were presented as mean ± SEM of 3 independent experiments. (DYM, ***p < 0.001 vs. circControl without RNase R; ^##^*p* < 0.01 vs. circDYM without RNase R using Student’s *t* test. circDYM, ***p < 0.001 vs. circControl without RNase R; ^###^*p* < 0.001 versus circControl with RNase R using Student’s *t* test). **e** Transduction with the circDYM-GFP lentivirus attenuated the iNOS expression induced by LPS in primary mouse microglia. Cells were transduced with circControl/circDYM-GFP lentivirus for 24 h and then treated with LPS (100 ng/ml) for another 24 h. All the data were presented as mean ± SEM of 3 independent experiments. (circDYM: F_(1,8)_ = 8.624, *P* < 0.05; LPS: F_(1,8)_ = 62.648, *P* < 0.001; interaction: F_(1,8)_ = 5.396, *P* < 0.05. **p < 0.01 versus circControl Control group; ^##^*p* < 0.01 versus circControl treated with LPS group). **f** circDYM was affinity-isolated with Bio-miR-9-WT or Bio-miR-9-mut. The Bio-miR-9-WT and Bio-miR-9-mut were incubated with HEK293T cell lysates at 25 °C for 2 h. All data were presented as mean ± SEM. ***p < 0.001 vs. the Bio-miR-9-WT GAPDH using Student’s *t* test. **g** miR-9 was affinity-isolated with a random probe or a circDYM probe. The biotinylated random and circDYM probe were incubated with HEK293T cell lysates at 25 °C for 2 h. All data were presented as mean ± SEM. ***p < 0.001 vs. circDYM probe U6 using Student’s *t* test. **h** Co-localization of circDYM and miR-9 in the cytoplasm of primary mouse microglia by FISH analysis. Green, circDYM; Red, miR-9; Blue, DAPI. Scale bar, 10 μm. **i** Transduction with the circDYM-GFP lentivirus significantly inhibited the iNOS expression induced by miR-9 in primary mouse microglia. All the data were presented as mean ± SEM of 3 independent experiments. (circDYM: F_(1,8)_ = 5.500, *P* < 0.05; miR-9: F_(1,8)_ = 58.883, *P* < 0.001; interaction: F_(1,8)_ = 5.972, *P* < 0.05. **p < 0.01 versus miR-Control transduced with circControl group; ^#^*p* < 0.05 versus miR-9 transduced with circControl group)

Consistent with in vivo findings, LPS treatment significantly decreased the expression of circDYM in both primary mouse microglia (Fig. 4a) and BV-2 cells (Supplementary Figure 8A). Next, microglia were transduced with the circDYM lentivirus to examine the role of circDYM in microglial activation. As expected, circDYM caused an increase in circDYM expression compared with circControl (Fig. 4b, Supplementary Figure 8B). To confirm that transduction with circDYM affects only circDYM, we treated RNA samples with RNase R, which cleaves linear RNAs. As shown in Fig. 4c, RNase R treatment resulted in a decreased level of linear DYM mRNA but not circDYM. However, transduction with circDYM increased the circDYM level in cells treated with RNase R, confirming that circDYM specifically enhanced circDYM expression (Fig. 4d). We further assessed the effect of circDYM on LPS-induced microglial activation. Transduction of cells with circDYM significantly inhibited LPS-induced microglial activation, as determined by the expression of iNOS in both primary mouse microglia (Fig. 4e) and BV-2 cells (Supplementary Figure 8C). Further examination indicated that overexpression of circDYM significantly inhibited the increased cytokines (IL-6, IL-1β, MCP-1, and TNF-α) induced by LPS in primary mouse microglia (Supplementary Figure 9).

Since miR-9 was predicted as a mediator of circDYM, their binding was tested via affinity isolation assay, demonstrated by the interaction between circDYM and miR-9. Furthermore, biotin-coupled miR-9 mimics were employed to assess whether miR-9 could bind with circDYM. More circDYM enrichment was detected in miR-9-captured fractions than in fractions in which mutations disrupted base-pairing between circDYM and miR-9 in HEK293T cells (Fig. 4f). This finding was confirmed by an inverse affinity isolation assay using a biotin-labeled specific circDYM probe (Fig. 4g).

FISH analysis confirmed that circDYM and miR-9 co-localized in the cytoplasm of primary mouse microglia (Fig. 4h). To verify that miR-9 is a mediator of circDYM, we co-transfected cells with miR-9 and circDYM. Overexpression of circDYM attenuated the inductive effect of miR-9 on microglial activation, as indicated by the iNOS expression in both primary mouse microglia (Fig. 4i) and BV-2 cells (Supplementary Figure 10). Further examination indicated that the overexpression of circDYM significantly inhibited the increased cytokines (IL-6, IL-1β, MCP-1, and TNF-α) induced by miR-9 in primary mouse microglia (Supplementary Figure 11).

### MiR-9 regulated microglial activation by targeting HECTD1

**Fig. 5:**
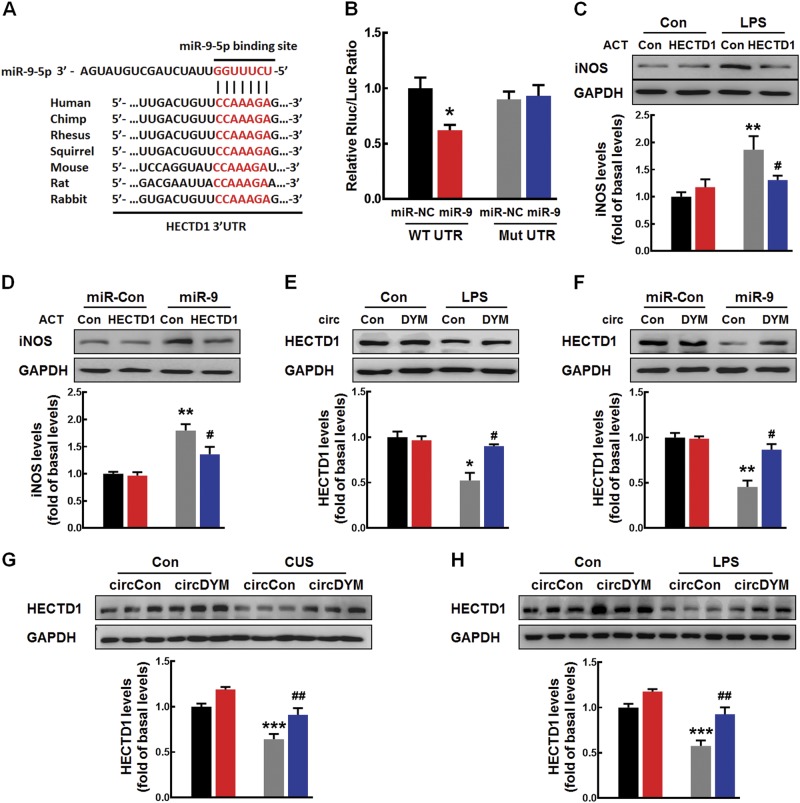
MiR-9 regulated microglial activation by targeting HECTD1. **a** Putative miR-9 binding sites in HECTD1 (HECTD1 gene). **b** Relative luciferase activity of wild-type and 3’-UTR mutant constructs of HECTD1 co-transfected with miR-9 mimics and miRNA negative control. The data were presented as mean ± SEM. *p < 0.05 versus miR-NC WT UTR using Student’s *t* test. **c** Transduction-ACT-upregulated HECTD1 significantly inhibited the increased iNOS expression induced by LPS in primary mouse microglia. All the data were presented as mean ± SEM of 3 independent experiments. (HECTD1-ACT: F_(1,8)_ = 5.391, *P* < 0.05; LPS: F_(1,8)_ = 29.975, *P* < 0.01; interaction: F_(1,8)_ = 11.766, *P* < 0.01. **P < 0.01 vs. Control-ACT Control group; ^#^*p* < 0.05 versus Control-ACT treated with LPS group). **d** Transfection of cells with HECTD1-ACT significantly inhibited the increased iNOS expression induced by miR-9 in primary mouse microglia. All data were presented as mean ± SEM of 3 independent experiments. (HECTD1-ACT: F_(1,8)_ = 7.627, *P* < 0.05; miR-9: F_(1,8)_ = 39.874, *P* < 0.001; interaction: F_(1,8)_ = 5.861, *P* < 0.05. **p < 0.01 vs. miR-Control transfected with Control-ACT group; ^#^*p* < 0.05 vs. miR-9 transfected with Control-ACT group). **e** Effect of circDYM overexpression on the decreased expression HECTD1 induced by LPS. Cells were transduced with the circControl/circDYM-GFP lentivirus for 24 h and then treated with LPS (100 ng/ml) for another 24 h in primary mouse microglia. All data were presented as mean ± SEM of 3 independent experiments. (circDYM: F_(1,8)_ = 8.703, *P* < 0.05; CUS: F_(1,8)_ = 21.364, *P* < 0.01; interaction: F_(1,8)_ = 12.326, *P* < 0.01. *p < 0.05 vs. circControl Control group; ^#^*p* < 0.05 versus circControl treated with LPS group). **f** circDYM overexpression significantly attenuated the decreased expression of HECTD1 induced by miR-9 in primary mouse microglia. All the data were presented as mean ± SEM of 3 independent experiments. (circDYM: F_(1,8)_ = 13.306, *P* < 0.01; miR-9: F_(1,8)_ = 36.924, *P* < 0.001; interaction: F_(1,8)_ = 14.735, *P* < 0.01. **p < 0.01 versus miR-Control transduced with circControl group; ^#^*p* < 0.05 versus miR-9 transduced with circControl group). **g**, **h** Effect of circDYM overexpression on the decreased expression of HECTD1 in the CUS (**g**) and LPS (**h**) model. Mice were microinjected with the circControl/circDYM-GFP lentivirus in the hippocampus. One week after microinjection, mice were exposed to a CUS protocol for 5 weeks or intraperitoneally injected with LPS (1 mg/kg) for 5 successive days. Three representative immunoblots were presented from 6 mice/group. All data were presented as mean ± SEM. (CUS model, circDYM: F_(1,20)_ = 50.088, *P* < 0.001; CUS: F_(1,20)_ = 46.113, *P* < 0.001; interaction: F_(1,20)_ = 5.085, *P* < 0.05. LPS model, circDYM: F_(1,20)_ = 36.839, *P* < 0.001; LPS: F_(1,20)_ = 48.185, *P* < 0.001; interaction: F_(1,20)_ = 5.034, *P* < 0.05). ***p < 0.001 versus circControl Control group; ^##^*p* < 0.01 vs. circControl treated with CUS/LPS group. Control-ACT Control CRISPR Activation Plasmid (ACT), HECTD1-ACT HECTD1 CRISPR Activation Plasmid (ACT)

Using the TargetScan algorithm, we predicted that a consensus binding site of miR-9 is present in the 3’-untranslated region (3’-UTR) of HECTD1. As shown in Fig. 5a, HECTD1 exhibits a conserved miR-9 binding site within its 3′-UTR in most species. This relationship was confirmed by luciferase assays. Co-transfection of an miR-9-overexpressing vector and the pmiR-GLO plasmid with the HECTD1 WT 3′-UTR resulted in the downregulation of luciferase activity, and this effect was reversed in HEK293T cells transfected with a mutated HECTD1 3′-UTR (Fig. 5b). Consistently, miR-9 decreased HECTD1 expression, whereas anti-miR-9 increased its expression in primary mouse microglia (Supplementary Figure 12A) and BV-2 cells (Supplementary Figure 12B). In line with this finding, LPS-treated mice exhibited significantly decreased expression of HECTD1 compared with the control group in both primary mouse microglia and BV-2 cells (Supplementary Figure 12C).

Having determined that miR-9 regulates HECTD1 expression, we next examined the role of HECTD1 in microglial activation induced by LPS. As shown in Supplementary Figure 12D, transfection with the HECTD1 CRISPR Activation Plasmid (ACT) upregulated the expression of HECTD1 in both primary mouse microglia and BV-2 cells. Moreover, transfection with HECTD1-ACT resulted in significant amelioration of the increased iNOS expression induced by LPS in both primary mouse microglia (Fig. 5c) and BV-2 cells (Supplementary Figure 13A). Further examination indicated that transfection with HECTD1-ACT significantly inhibited the increased cytokines (IL-6, IL-1β, MCP-1, and TNF-α) induced by LPS in primary mouse microglia (Supplementary Figure 13B-E).

In order to examine the functional relationship between miR-9 and HECTD1, we transfected cells with HECTD1-ACT followed by an examination for microglial activation. Transfecting cells with HECTD1-ACT significantly inhibited the increased iNOS expression induced by miR-9 in both primary mouse microglia (Fig. 5d) and BV-2 cells (Supplementary Figure 14A). Further examination indicated that transfection with HECTD1-ACT significantly inhibited the increased cytokines (IL-6, IL-1β, MCP-1, and TNF-α) induced by miR-9 in primary mouse microglia (Supplementary Figure 14B-E).

We next examined the role of circDYM in regulating the expression of HECTD1. Overexpression of circDYM significantly ameliorated the decreased expression of HECTD1 induced by LPS treatment in both primary mouse microglia (Fig. 5e) and BV-2 cells (Supplementary Figure 15A). In contrast, circDYM reversed the decrease in HECTD1 expression induced by miR-9 overexpression in both primary mouse microglia (Fig. 5f) and BV-2 cells (Supplementary Figure 15B). These results indicated that circDYM acted as an endogenous miR-9 sponge to regulate HECTD1 expression. The in vivo relevance was confirmed, as microinjection of the circDYM lentivirus significantly inhibited the decreased expression of HECTD1 in CUS and LPS mice (Fig. 5g, h).

### CircDYM/miR-9/HECTD1 regulated microglial activation via HSP90 ubiquitination in vitro

HECTD1 is involved in ubiquitination because it encodes a novel protein homologous to the E6-AP C-terminal (HECT) domain-containing E3 ubiquitin (Ub) ligase [39]. Since HSP90 is the substrate of ubiquitination mediated by HECTD1 and is involved in microglial activation, we examined the effect of LPS on HSP90 ubiquitination. As shown in Supplementary Figure 16A-B, LPS treatment of BV-2 cells decreased HSP90 ubiquitination as well as K63 ubiquitination. Further examination demonstrated that HSP90 expression increased after LPS treatment in both primary mouse microglia and BV-2 cells (Supplementary Figure 16C). In addition, an immunoprecipitation assay revealed an interaction between HECTD1 and HSP90 (Supplementary Figure 16D) as well as between HSP90 and K63 Ub (Supplementary Figure 16E). To examine whether HECTD1 is involved in HSP90 degradation via ubiquitination, we performed immunoprecipitation experiments using HECTD1-ACT. As shown in Supplementary Figure 16F, HECTD1-ACT enhanced the interaction between HSP90 and K63-ubiquitin, indicating that HECTD1-mediated HSP90 expression occurred via ubiquitination.

Next, we examined the role of circDYM in LPS induced ubiquitination. As shown in Supplementary Figure 16G, transduction of cells with the circDYM lentivirus significantly inhibited the decreased K63-ubiquitin expression induced by LPS. Consistently, the increased level of HSP90 was significantly ameliorated by circDYM (Supplementary Figure 16H). HECTD1-ACT inhibited the increased expression of HSP90 induced by LPS treatment (Supplementary Figure 16I). Downstream, inhibition of HSP90 significantly reduced the increase in iNOS expression induced by LPS (Supplementary Figure 16J).

### CircDYM regulated microglial activation via HSP90 ubiquitination in vivo

On the basis of our in vitro findings, we further explored the role of circDYM in HSP90 ubiquitination in vivo. As shown in Supplementary Figure 17A-B, CUS mice had increased HSP90 expression and reduced K63 ubiquitination in the hippocampus compared with the control group. Moreover, overexpression of circDYM significantly attenuated decreased HSP90 ubiquitination as determined by immunoprecipitation using HSP90 followed by examination for K63 ubiquitination (Supplementary Figure 17C). These findings were confirmed in the LPS-treated group (Supplementary Figure 17D-F).

## Discussion

Our study demonstrated that circDYM is downregulated in both the peripheral blood of MDD patients and the depressive-like animal models. Decreased expression of circDYM released miR-9 with concomitant downstream downregulation of HECTD1, resulting in the inhibition of HSP90 ubiquitination and the increase of microglial activation (Supplementary Figure 18). Upregulation of circDYM may thus represent a potential therapeutic strategy for depression. This study highlights the role of circRNAs in MDD and provides new insight into the development of potential preventive strategies and effective treatment for MDD.

To our knowledge, this is the first study to report that circDYM is a potential therapeutic target for MDD. The level of circDYM was significantly reduced in the peripheral blood of MDD subjects and in the hippocampus of depressive animal models. The restoration of circDYM in the brain of depressive animal models ameliorated depressive-like behavior. Although it is unclear how circRNA levels in the periphery and the brain interact during MDD, given their consistent dysregulation, it is possible that circRNAs may actively cross the blood-brain barrier. Indeed, circRNAs appear to modulate both immune and neuronal processes and may mediate the interaction between these systems. In MDD subjects, levels of circDYM in the peripheral blood are negatively correlated with the TEPS-A score, which is an effective measurement for anhedonia, a core clinical symptom of MDD. Our study found that circDYM and childhood trauma events had an interactive effect on the severity of anhedonia symptoms in MDD patients, indicating that both biological and environmental factors play a role in the occurrence of depression. Together, our results suggest that dysregulation of circDYM in peripheral blood is associated with the pathophysiology of MDD.

Although miRNAs are considered potential biomarkers of MDD and treatment response [7, 40], the role of miR-9 in MDD is largely unknown. The present study indicated that the level of miR-9 is significantly increased in MDD subjects and is positively associated with HAMA scores and anxiety symptoms. Our study also showed that miR-9 and childhood trauma events could play an interactive effect on anhedonia symptoms in MDD patients, suggesting that miR-9 is associated with the pathophysiology of MDD. Accumulating evidence indicates that miRNAs are involved in microglial function. miR-124 is involved in microglial polarization [41], and other miRNAs, including miR-let-7c-5p, miR-145-5p, miR-155, and miR-125b, are involved in microglial activation in different neuronal disorders [42–45]. In the context of depression, our previous work indicated that miR-9 regulates microglial activation induced by LPS, which is consistent with a recent study showing that circulating miR-9 is upregulated in the prefrontal cortex of a CUS model [24]. However, another study indicated that CUS decreased the expression of miR-9 in the nucleus accumbens and striatum [25]. This discrepancy may be caused by different stimulations in different species. Regarding of schizophrenia, previous study indicated that schizophrenia patients showed statistically significant upregulation of miR-9 in the peripheral blood [46]. However, Topol et al demonstrated that the level of miR-9 was decreased in the subset of schizophrenia-patient-derived neural progenitor cells [47]. The specific correlation of miR-9 and these diseases need to be confirmed in larger post-mortem cohorts or sample sizes.

In the present study, the interaction between miR-9 and circDYM was confirmed via FISH and affinity isolation assays. Thus, circRNAs may act as an endogenous miR-9 sponge to regulate its target gene expression and thereby ameliorate depressive-like behavior. These findings are consistent with previous studies where it was found that the circRNA HRCR acts as a miR-223 sponge to regulate cardiac hypertrophy and heart failure [48] and another circRNA, SRY, acts as a sponge for miR-138 [49]. In addition to regulating miRNAs, circRNAs exert their biological functions via other mechanisms. For example, circFox3 was found to participate in cell cycle progression by forming a ternary complex with p21 and CDK2 [15]. Another complex, circRNA-cZNF292, is regulated by hypoxia and displays pro-angiogenic activities in endothelial cells. Intriguingly, some circRNAs may not solely function as non-coding RNAs. circRNAs circ-ZNF609 has been reported to translate proteins through a splicing-dependent, cap-independent mechanism and function in myogenesis [50]. We demonstrated that miR-9 is regulated by circDYM; however, we could not rule out the possibility that other mechanisms may underlie the functions of circDYM.

Having demonstrated that circDYM/miR-9 regulated microglial alteration during depression, we further examined the downstream target of miR-9 and the mechanism underlying miR-9-mediated microglial activation. Computational algorithms such as TargetScan have been employed in previous studies to identify evolutionarily conserved sequences in HECTD1 that are targeted by miR-9. Consistent with TargetScan predictions, our findings indicated that HECTD1 was a target of miR-9 based on Luciferase activity assay results. The gene encoding HECTD1 has been mapped to a region of chromosome 14q12 encoding a novel protein homologous to the E6-AP C-terminal (HECT) domain-containing E3 ubiquitin ligase, which regulates the selective ubiquitination of HSP90 client proteins [39, 51]. Consistent with these findings, our study indicated that HSP90 was the target for HECTD1 ubiquitination. Together with previous studies [52, 53], we showed that inhibition of HSP90 significantly decreased microglial activation. We also demonstrated that HECTD1, as a target of miR-9, functions in the microglial activation induced by CUS and LPS in vivo. The detailed mechanism of how HSP90 ubiquitination by HECTD1 is involved in this process will be further examined in future studies.

Chronic stress dynamically affects microglial activity, inducing initial activation and proliferation followed by apoptosis, dystrophy, and decline [30, 31]. Acute stressors induce microglial activation in many brain regions [34, 54], and the effects of chronic stress on microglial activation are heterogeneous due to multiple variables, including the stress paradigm (timing and types of stressors) [30, 55–58] and the age of animals [59]. Consistent with previous studies [55–58], the current study demonstrated robust and persistent effects of CUS on microglial morphology.

Taken together, findings of this study demonstrated for the first time that circDYM binds with miR-9 and acts as an endogenous sponge to inhibit miR-9 activity, resulting in increased expression of HECTD1 and HSP90 ubiquitination with the consequent inhibition of microglial activation. Our study provides proof-of-concept evidence that restoration of circDYM attenuated depressive-like behavior through the regulation of microglial dysfunction. Thus, circDYM may be important as a promising target for therapeutic interventions in MDD.

## Electronic supplementary material

Supplementary Information
