# Supplementary material for: CircDYM ameliorates depressive-like behavior by targeting miR-9 to regulate microglial activation via HSP90 ubiquitination
Source: Mol Psychiatry. 2018 Nov 9;25(6):1175–90. doi: 10.1038/s41380-018-0285-0 (PMC7244405; doi:10.1038/s41380-018-0285-0)
Supplement: Supplementary file 1 — Supplementary Information [file 41380_2018_285_MOESM1_ESM.docx]

**CircDYM ameliorates depressive-like behavior by targeting miR-9 to regulate microglial activation via HSP90 ubiquitination**

Yuan Zhang^1†^, Longfei Du^1†^, Ying Bai^1†^, Bing Han^1†^, Cancan He^2†^,Liang Gong^2^, Rongrong Huang^1^, Ling Shen^1^,Jie Chao^3^, Pei Liu^4^, Hongxing Zhang^5,6^,Haisan Zhang^6^, Ling Gu^7^, Junxu Li^8^, Gang Hu^9^, Chunming Xie^2*^, Zhijun Zhang^2,5,6*^, Honghong Yao^1,10*^

**Abbreviated title: Role of circDYM in depression**

^1^Department of Pharmacology, School of Medicine, Southeast University, Nanjing, 210009, Jiangsu, China; ^2^Department of Neurology of Affiliated ZhongDa Hospital, Institute of Neuropsychiatry of Southeast University, Nanjing, 210029, Jiangsu, China; ^3^Department of Physiology, School of Medicine, Southeast University, Nanjing, 210009, Jiangsu, China; ^4^Department of Epidemiology and Biostatistics, School of Public Health, Southeast University, Nanjing, 210029,Jiangsu, China; ^5^Department of Psychology of Xinxiang Medical University, Xinxiang, Henan, 453003, China; ^6^Second Affiliated Hospital of Xinxiang Medical University, Xinxiang, Henan, 453003, China; ^7^Basic Medical College, Nanjing University of Chinese Medicine, Nanjing, 210046, Jiangsu, China; ^8^Department of Pharmacology and Toxicology, University at Buffalo, Buffalo, NY, USA; ^9^Jiangsu Key Laboratory of Neurodegeneration, Department of Pharmacology, Nanjing Medical University, Nanjing, 210029, Jiangsu, China; ^10^Institute of Life Sciences, Key Laboratory of Developmental Genes and Human Disease, Southeast University, Nanjing, 210096, Jiangsu, China

† These authors contributed equally to this work.

*To whom correspondence should be addressed:

Honghong Yao, Ph.D., Department of Pharmacology, Medical School of Southeast University, Nanjing, 210009, Jiangsu, China; Tel: +8625 83272551; E-mail: yaohh@seu.edu.cn

Zhijun Zhang, Ph.D., Department of Neurology of Affiliated ZhongDa Hospital, Institute of Neuropsychiatry of Southeast University, Nanjing, 210029, Jiangsu, China; Tel: +86 25 83262241; E-mail: janemengzhang@vip.163.com

Chunming Xie, Ph.D., Department of Neurology of Affiliated ZhongDa Hospital, Institute of Neuropsychiatry of Southeast University, Nanjing, 210029, Jiangsu, China; Tel: +86 25 83262253; E-mail: chmxie@163.com

**Table of Contents**

Supplementary Materials and Methods……………………………………………3

References………………………………………….………………………………12

Supplementary Figures S1-S18………………………………………………….13

Supplementary Tables S1-S5…………………………………………………….33

**Supplementary Materials and Methods**

**Inclusion and Exclusion Criteria**

For individuals in the MDD group, subjects were required to meet the following criteria: (1) meet the diagnostic criteria for MDD using a Structured Clinical Interview by two neuropsychiatrists (C Xie and HX Zhang) according to the Diagnostic Statistical Manual of Mental Disorder, Fifth Edition (DSM-V); (2) the scores larger than 17 which was measured by Hamilton Depression Rating Scale (HAMD-17); (3) drug naïve or drug free for longer than three weeks; (4) aged between 18 and 59 with the age of MDD onset under 55. Exclusion criteria for the MDD group included the following: (1) other major psychiatric disorder or neurodegenerative disease history and (2) substance abuse, head trauma, or loss of consciousness.

Healthy control subjects were required to have a HAMD-17 score ≤ 7 and a Montreal Cognitive Assessment (MoCA) score ≥ 26. Exclusion criteria include history of neuropsychiatric disease, head injury, drug abuse or insobriety.

**Behavior Measurements**

All subjects were given a comprehensive clinical assessment of their neurological and mental status. HAMD was used to obtain a depression evaluation. The following scales were also applied for assessment including Temporal Experience of Pleasure Scale (TEPS), Physical Anhedonia Scale (PAS), Social Anhedonia Scale (SAS), Hamilton Anxiety Scale (HAMA), Regulatory Focus Questionnaire (RFQ) and Childhood Trauma Questionnaire (CTQ).

**Sucrose preference test (SPT)**

As previously described [^1^](#_ENREF_1), SPT is conducted in three phases as follows: phase 1 habituation, phase 2 sucrose preference baseline, and phase 3 sucrose preference testing. In phase 1, 1% w/v sucrose solution was used in the cage for 3 days to habituate mice to the solution. In phase 2, each mouse was transferred to a single cage and was exposed to both tap water and sucrose solution for 24 h to obtain the sucrose preference baseline. Sucrose preference was then tested via a two-bottle choice test using standard bottles, one filled with tap water and one with 1% sucrose solution, supplied to mice for 24 h (phase 3). The locations of the bottles (left or right) were counterbalanced across the study. Tap water and sucrose solution intake was quantified by subtracting the final weight of bottles after 24 h exposure from their initial weight. The preference to consume sucrose solution was then calculated as percentage preference = [(sucrose intake/total intake) × 100]. Tests were performed by an individual blind to the animal’s treatment status.

**Forced swim test (FST)**

FST was carried out as described elsewhere with slight modifications [^2^](#_ENREF_2)^,^ [^3^](#_ENREF_3). Mice were dropped individually into a cylinder (diameter: 20 cm; height: 25 cm) filled with 15 cm water maintained at 23-25 °C. After vigorous activity in the first 2 min, mice acquired an immobile posture, which was characterized by motionless floating in the water with only necessary movements to keep their heads above water. The duration of immobility was recorded during the last 4 min of the 6 min test. Tests were performed by an individual blind to the animal’s treatment status.

**Tail suspension test (TST)**

TST was considered to mirror despair/depression-like behavior by analysis of the immobility time during the test. Mice were suspended 50 cm above the floor by adhesive tape placed approximately 1 cm from the tip of the tail in the apparatus box (50 × 50 × 50 cm). Each test session lasted 6 min and was recorded. The first 2 min served as the habituation period. During the last 4 min, duration of immobility (hanging passively without body movement) was measured. Tests were performed by an individual blind to the animal’s treatment status.

**Immunostaining**

As described in our previous study [^4^](#_ENREF_4), tissue sections of 30 μm were prepared with a cryostat. The sections were incubated with H_2_O_2_ for 10 min, incubated with 0.3% Triton X-100 in phosphate-buffered saline (PBS; 137 mM NaCl, 2.7 mM KCl, 10 mM Na_2_HPO_4_, 2 mM KH_2_PO_4_) for 15 min, and then blocked with 10% normal goat serum (NGS) in 0.3% Triton X-100 for 1 h at room temperature. Next, the sections were incubated with a rabbit anti-Iba-1 antibody (1:250, Wako, 019-19741) overnight at 4°C. On the following day, the sections were washed, incubated with biotinylated goat anti-rabbit IgG (Vector Laboratories, BA-1000) in PBS for 1 h at room temperature, and then incubated with VECTASTAIN^®^ (VECTASTAIN^®^ ABC Kit, Vector Laboratories, PK-6200) for 1 h. The horseradish peroxidase reaction product was visualized using an enhanced DAB peroxidase substrate kit (Vector Laboratories, SK-4100).

**Western blotting (WB)**

As in our previous study [^5^](#_ENREF_5), proteins were extracted in RIPA lysis buffer (Beyotime, P0013B), separated on sodium dodecyl sulfate polyacrylamide gels (12% and 15%), and electrophoretically transferred onto polyvinylidene fluoride membranes. The membranes were blocked with 5% non-fat dry milk in Tris-buffered saline with 0.2% Tween-20; probed with antibodies recognizing HECTD1 (1:1000, Proteintech, 20605-1-AP), iNOS (1:100, Proteintech, [18985-1-AP](https://www.ptglab.com/products/NOS2-Antibody-18985-1-AP.htm)), HSP90 (1:1000, Proteintech, [13171-1-AP](https://www.ptglab.com/products/HSP90-Antibody-13171-1-AP.htm)), Ub63 (1:1000, abways, CY6579), and GAPDH (1:1000, Proteintech, [60004-1-Ig](https://www.ptglab.com/products/GAPDH-Antibody-60004-1-Ig.htm)) overnight at 4°C; and then incubated with a horseradish peroxidase-conjugated goat anti-mouse/rabbit IgG secondary antibody (1:2000, Cell Signaling, 7076P2/7074P2). Signals were detected by chemiluminescence and imaged on a Microchemi 4.2^®^ (DNR, Israel) digital image scanner. Quantification of the individual protein bands was performed by densitometry using Image J software (NIH, USA).

**Real-time PCR**

Real-time PCR for mature miR-9 was performed according to our previous studies in an Applied Biosystems Real-time PCR System [^5^](#_ENREF_5). First, total RNA was extracted using TRIzol reagent (Invitrogen, 15596026) and treated with gDNA wiper. Then, the RNA was reverse transcribed with a stem-loop RT primer (RiboBio, Guangzhou, China) using a HiScript Q Select RT SuperMix for qPCR Kit (Vazyme, R133-01). Next, the RT products were quantified using AceQ qPCR SYBR Green Master Mix (Vazyme, R141-02). The levels of miR-9 analyzed by real-time PCR were normalized to that of U6. Specific primers for mature miR-9 and U6 were obtained from RiboBio (Guangzhou, China). circDTM was were reverse transcribed using a HiScript Q RT SuperMix for qPCR Kit (Vazyme, R123-01) and quantified using SYBR Green Real-time PCR Master Mix. The results were standardized to control values of GAPDH. Blind detection was done by two investigators (LF Du and B Han) blinded to the group allocations.

**Fluorescence in situ hybridization (FISH)**

As described in our previous studies [^5^](#_ENREF_5)^,^ [^6^](#_ENREF_6), primary mouse microglia cultured on coverslips were fixed with 4% PFA for 20 min, incubated in PBS overnight at 4°C, and processed to detect circDYM or miR-9 expression. The cells were permeabilized with 0.25% Triton X-100 in PBS for 15 min and pre-hybridized in hybridization buffer (50% formamide, 10 mM Tris-HCl, pH 8.0, 200 μg/ml yeast tRNA, 1 x Denhardt's solution, 600 mM NaCl, 0.25% SDS, 1 mM EDTA, and 10% dextran sulfate) for 1 h at 37°C. Then, the coverslips were heated to 65°C for 5 min in hybridization buffer containing 50 nM of a commercially available biotin-labeled circDYM probe or 25 nM of a commercially available digoxigenin-labeled miR-9 probe. Hybridization was allowed to occur at 37°C overnight. The next day, the coverslips were washed three times in 2 x SSC and twice in 0.2 x SSC at 42°C, blocked with a solution of 1% BSA and 3% NGS in PBS for 1 h at room temperature, and then incubated with a horseradish peroxidase-conjugated anti-digoxigenin antibody (1:200, Roche, 11207733910) and FITC-Streptavidin (1:200, Invitrogen, 434311) overnight at 4°C. After the coverslips were washed three times with TBS, signal amplification was carried out using a TSA Cy5 kit (PerkinElmer, NEL745001KT) for 10 min at room temperature. Then, the coverslips were washed twice with PBS and then incubated with Hoechst 33342 (Invitrogen, H1399) for 1 min at room temperature to visualize the nuclei. Finally, the sections were washed once with DEPC water and mounted with 30% glycerine. The images were captured via microscopy (ZEISS, Oberkochen, Germany, LSM700). The mouse circDYM probe sequence, which was biotinylated at the 5' end, was 5'-AAACGAGGGTTGTTTTCAAAAGAGTGGAGTATCAG-3'. The 5' digoxigenin-labeled miR-9 probe sequence was 5'-TCATACAGCTAGATAACCAAAGA-3'. These probes were all synthesized by Invitrogen.

**FISH in combination with immunostaining**

As described in our previous studies [^5^](#_ENREF_5)^,^ [^6^](#_ENREF_6), brain sections encompassing the entire hippocampus were cut into 30 μm sections using a cryostat. The sections were fixed in 4% PFA for 20 min and washed twice with PBS. The sections were permeabilized with 0.3% Triton X-100 in PBS for 15 min and pre-hybridized in hybridization buffer for 1 h at 37°C. Hybridization buffer with 50 nM of a commercially available biotin-labeled circDYM probe was heated to 65°C for 5 min and added dropwise to the sections, which were then allowed to hybridize at 37°C overnight. The next day, the sections were washed three times in 2 x SSC and twice in 0.2 x SSC at 42°C. The sections were blocked with 1% BSA and 3% normal goat serum in PBS for 1 h at room temperature and then incubated with FITC-Streptavidin (1:200, Invitrogen, 434311) overnight at 4°C. On the third day, after the sections were washed three times with PBS, they were blocked with a solution of 1% BSA and 1% Triton X-100 in PBS for 1 h at room temperature and then incubated with a rabbit anti-Iba-1 antibody (1:250, Wako, 019-19741) overnight at 4°C. On the fourth day, after the sections were washed three times with PBS, they were incubated with Alexa-Fluor 594 goat anti-rabbit IgG (1:250, Invitrogen, A11012) for 1 h at room temperature. The samples were washed twice in PBS and once in DEPC water and mounted with Prolong gold anti-fade reagent containing DAPI. Immunofluorescence images were captured using confocal microscopy (ZEISS, Oberkochen, Germany, LSM700).

**Cell cultures**

As described in our previous study [^5^](#_ENREF_5), primary mouse microglia cells were obtained from postnatal (P1 to P2) C57BL/6J mice, which were purchased from the Comparative Medicine Center, Yangzhou University. After the membranes and large blood vessels were dissociated, the dissected brain cortices in PBS supplemented with brain tissues were digested with trypsin-EDTA (Gibco, 25200056). Cells were seeded in 25 cm^2^ cell culture flasks which pre-coated with poly-D-lysine (SigmaAldrich, P0296). Cells were cultured in Dulbecco’s modified Eagle’s medium (DMEM) supplemented with fetal bovine serum (10% v/v) and penicillin-streptomycin (1% v/v). After 3 d, the medium was changed for the first time. Seven days later, the medium was replaced every 3 d, and CSF2/GM-CSF (colony stimulating factor 2 [granulocyte-macrophage]; 0.25 ng/ml; PeproTech, 315-03) was added to the flasks to promote microglial proliferation. The microglia were detached from the flasks by shaking and collected from the cell medium by centrifugation at 1500 g for 5 min. BV-2 cells were obtained from the China Center for Type Culture Collection, routinely maintained in DMEM (10% fetal bovine serum, 1% penicillin-streptomycin) and incubated in 5% CO_2_ at 37°C. Cell line was authenticated and tested negative for mycoplasma contamination.

**Transduction of microglia with lentivirus**

As described in our previous study [^7^](#_ENREF_7), microglia were transduced with the circControl or circDYM lentivirus (Hanbio, Shanghai, China) at a multiplicity of infection of 1 (primary mouse microglia) or 10 (BV-2 cells), followed by gentle swirling, incubation, and replacement of fresh feed medium.

**Luciferase activity assays**

As described in our previous study [^5^](#_ENREF_5), the 3'-UTR of the 723-bp human *HECTD1* gene containing the putative *miR-9* target site was PCR amplified from human genomic DNA using forward (5’-GCGGCTCGAGTGCAAGCTATCCATCAGTC-3’) and reverse (5’-AATGCGGCCGCATAGTATGGCATTTAGGAA-3’) primers, and the DNA fragment was cloned into the XhoI and NotI sites at the 3'-end of the *luc2* gene in the pmiR-RB-REPORT^TM^ vector (RiboBio, Guangzhou, China). For the pmiR-RB-*HECTD1*-3'-UTR-*miR-9*-target-mutant vector, the *miR-9* target site (CCAAAGA) within the *HECTD1* 3'-UTR was changed to GGTTTCT via PCR mutagenesis with the primers *HECTD1*-*miR-9*-F (5’-TGACTGTTGGTTTCTGCAGCTTCTCAGATCTTC-3’) and *HECTD1*-*miR-9*-R (5’-GAAGCTGCAGAAACCAACAGTCAAGTGGGACCA-3’). Briefly, HEK293T cells were transfected with a miR-9 mimic (RiboBio, Guangzhou, China) and a target plasmid, pmiR-RB-*HECTD1*-3'-UTR or pmiR-RB-*HECTD1*-3'-UTR-*miR-9*-target-mutant, at a molar ratio of 50:1. A miRNA control was used as a negative control. Luciferase activity was determined 24 h post-transfection, and reporter assays were performed following the manufacturer’s protocol (Promega, E2920). Renilla luciferase activity was normalized to firefly luciferase activity and expressed as a percentage of the control.

**Enzyme-linked immunosorbent assay (ELISA)**

Cell culture supernatant was collected by centrifugation for 15 min at 1000 g, 4°C. The tissues of hippocampus (100 mg) was rinsed and homogenized in PBS (1 ml) and stored overnight at -20°C. After two freeze-thaw cycles were performed to break the cell membranes, the homogenates were centrifuged for 5 min at 5000 g, 4°C and the supernatant was collected. The levels of cytokines (IL-6, IL-1β, MCP-1 and TNF-α) in the cell culture supernatant and hippocampal extracts was analyzed using commercially available ELISA kits, according to manufacturer’s instructions. The ELISA Kit of Mouse IL-6 (JEB-12267), Mouse IL-1β (JEB-12787) and Mouse MCP-1(JEB-15205) were purchased from Nanjing Jin Yibai Biological Technology Co. Ltd (Nanjing, China), and the Mouse TNF-α ELISA Kit (DY410-05) was from R&D Systems (Minneapolis, MN, USA).

**References**

1. Willner P, Towell A, Sampson D, Sophokleous S, Muscat R. Reduction of sucrose preference by chronic unpredictable mild stress, and its restoration by a tricyclic antidepressant. *Psychopharmacology* 1987; **93**(3)**:** 358-364.

2. Porsolt RD, Bertin A, Jalfre M. Behavioral despair in mice: a primary screening test for antidepressants. *Archives internationales de pharmacodynamie et de therapie* 1977; **229**(2)**:** 327-336.

3. Ramamoorthy R, Radhakrishnan M, Borah M. Antidepressant-like effects of serotonin type-3 antagonist, ondansetron: an investigation in behaviour-based rodent models. *Behavioural pharmacology* 2008; **19**(1)**:** 29-40.

4. Yao H, Ma R, Yang L, Hu G, Chen X, Duan M *et al.* MiR-9 promotes microglial activation by targeting MCPIP1. *Nature communications* 2014; **5:** 4386.

5. Zhang Y, Shen K, Bai Y, Lv X, Huang R, Zhang W *et al.* Mir143-BBC3 cascade reduces microglial survival via interplay between apoptosis and autophagy: Implications for methamphetamine-mediated neurotoxicity. *Autophagy* 2016; **12**(9)**:** 1538-1559.

6. Bai Y, Zhang Y, Han B, Yang L, Chen X, Huang R *et al.* Circular RNA DLGAP4 Ameliorates Ischemic Stroke Outcomes by Targeting miR-143 to Regulate Endothelial-Mesenchymal Transition Associated with Blood-Brain Barrier Integrity. *The Journal of neuroscience : the official journal of the Society for Neuroscience* 2018; **38**(1)**:** 32-50.

7. Huang R, Zhang Y, Han B, Bai Y, Zhou R, Gan G *et al.* Circular RNA HIPK2 regulates astrocyte activation via cooperation of autophagy and ER stress by targeting MIR124-2HG. *Autophagy* 2017; **13**(10)**:** 1722-1741.

**Supplementary Figures**

**
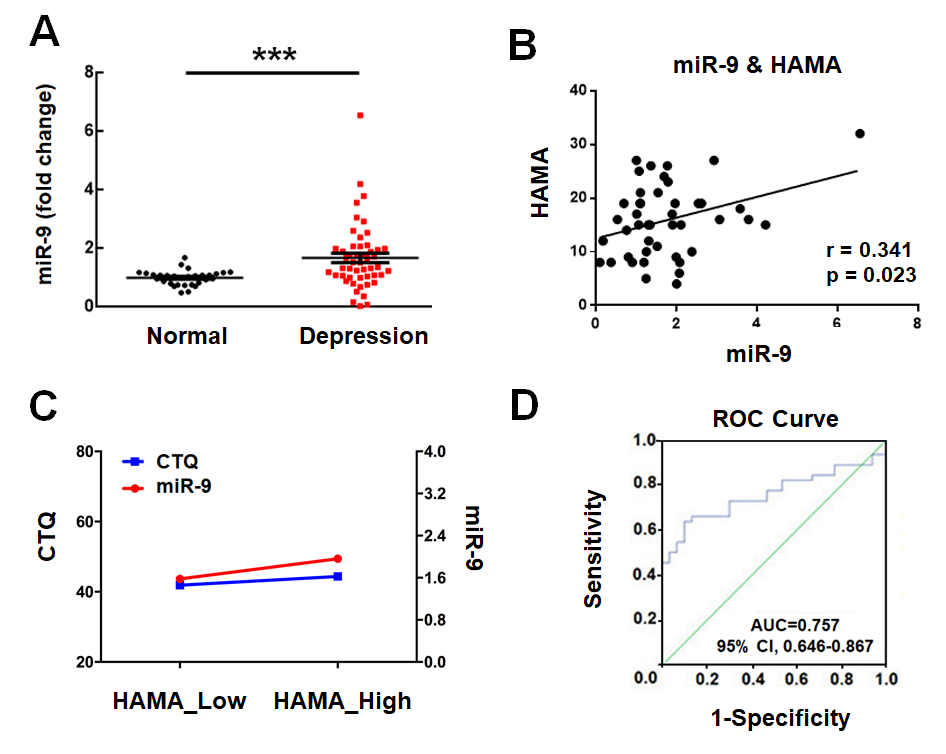
**

**Supplementary Figure 1. miR-9 was up-regulated in the serum of MDD patients. (A)** miR-9 expression levels in the serum of MDD patients were increased compared with those in normal controls. All data were presented as mean ± SEM. n=30 individuals/control group; n=44 individuals/MDD group. ***p<0.001 versus Normal using Mann-Whitney U test. **(B)** Correlation between miR-9 expression and HAMA using Pearson’s correlation coefficient. **(C)** The interactive effect of miR-9 and CTQ on the core symptom of depression in MDD patients using Multivariate Linear Regression. **(D)** ROC curve for individual miR-9 to separate MDD patients from normal controls.


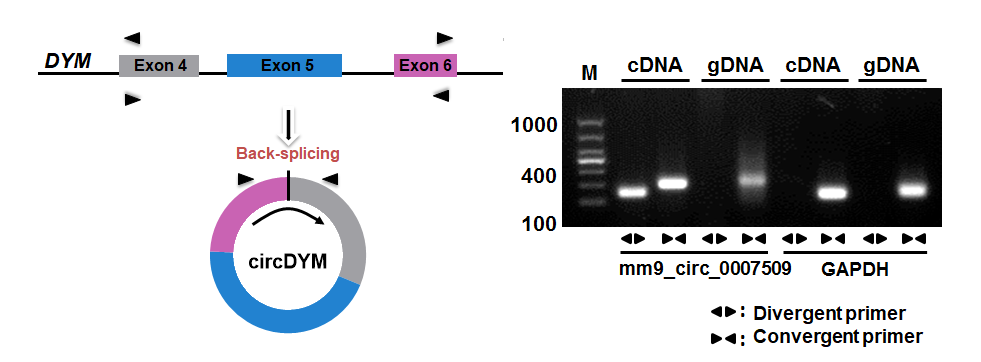


**Supplementary Figure 2. Divergent primers specific to circDYM.** CircDYM was derived from exon 4, 5, and 6 as illustrated in the left panel. Divergent primers amplified circDYM from cDNA but not genomic DNA (gDNA) as illustrated in the right panel. GAPDH, linear control. M, marker.


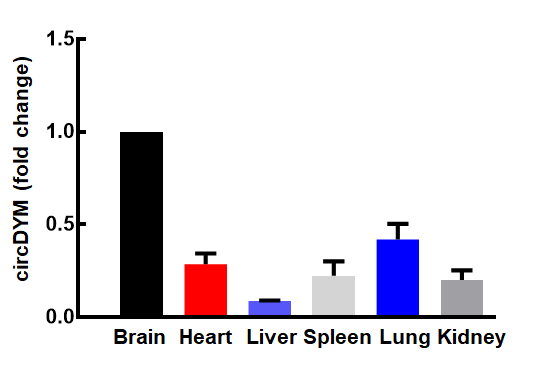


**Supplementary Figure 3. Expression of circDYM in different organs.** Expression of circDYM in the mouse brain, heart, liver, spleen, lung, and kidney.

**
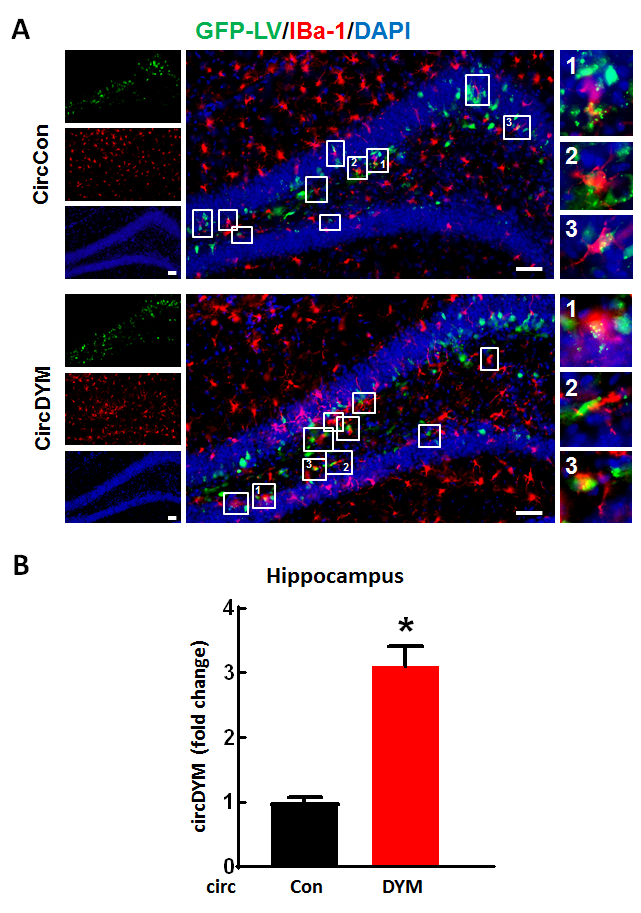
**

**Supplementary Figure 4. Overexpression of circDYM in hippocampus. (A)** Representative images of C57BL/6J mice microinjected with either circControl or circDYM-GFP lentivirus (1 μl of 1×10^9^ viral genomes/µl) into the hippocampus. Scale bar: 50 μm. **(B)** Expression of circDYM in the hippocampus of mice microinjected with lentivirus was determined by real-time PCR. All data were presented as mean ± SEM. n=6 mice/group, *p<0.05 versus circControl using Student’s t-test.

**
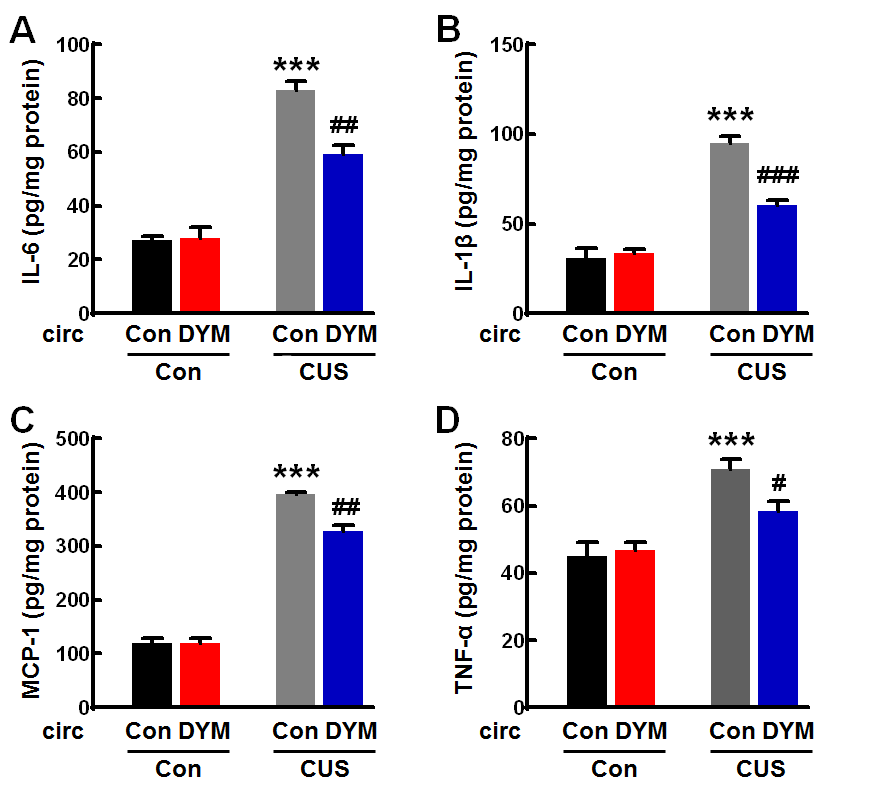
**

**Supplementary Figure 5.** **Overexpression of circDYM inhibited the expression of cytokines in the hippocampus induced by CUS.** **(A-D)** Mice were microinjected with the circControl/circDYM-GFP lentivirus in the hippocampus. One week after microinjection, mice were exposed to CUS for 5 weeks. The tissues of hippocampus was rinsed and homogenized in PBS. After centrifugation, the supernatant ﬂuids were collected and assayed by ELISA assay for production of IL-6 **(A)**, IL-1β **(B)**, MCP-1 **(C)** and TNF-α **(D)**. All data were indicated as mean ± SEM. n=6 mice/group. (IL-6, circDYM: F_(1,20)_=9.722, P<0.01; CUS: F_(1,20)_=136.849, P<0.001; interaction: F_(1,20)_=11.246, P<0.01. IL-1β, circDYM: F_(1,20)_=20.366, P<0.001; CUS: F_(1,20)_=137.818, P<0.001; interaction: F_(1,20)_=21.372, P<0.001. MCP-1, circDYM: F_(1,20)_=17.588, P<0.001; CUS: F_(1,20)_=840.932, P<0.001; interaction: F_(1,20)_=17.388, P<0.001. TNF-α, circDYM: F_(1,20)_=4.547, P<0.05; CUS: F_(1,20)_=37.356, P<0.001; interaction: F_(1,20)_=4.515, P<0.05. ***p<0.001 versus circControl Control group; ^#^p<0.05, ^##^p<0.01 and ^###^p<0.001 versus circControl induced by CUS group).


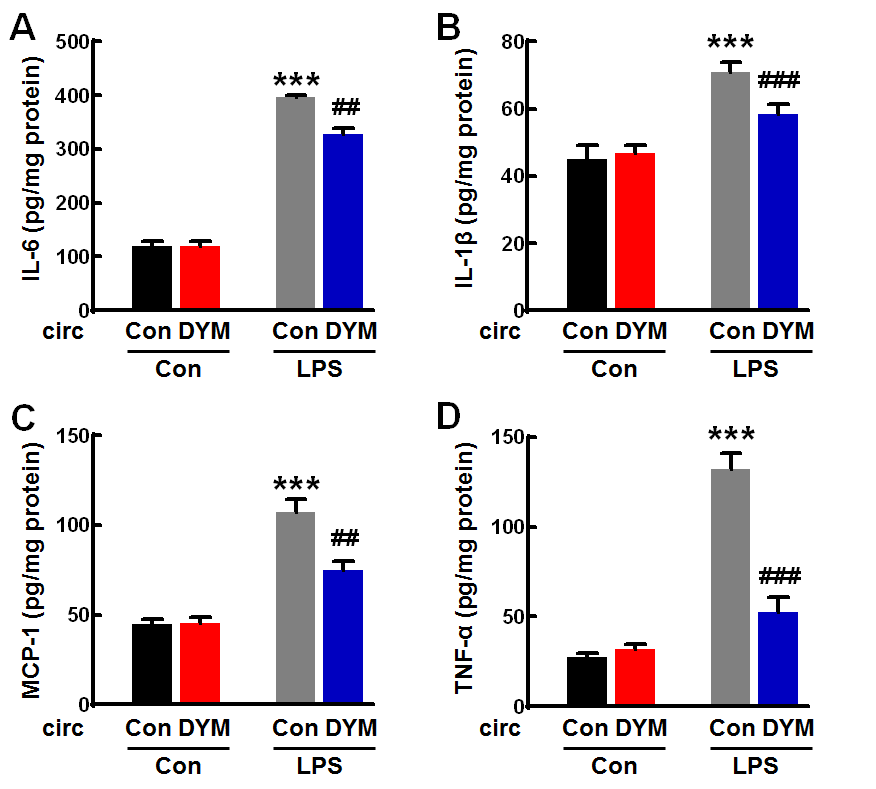


**Supplementary Figure 6.** **Overexpression of circDYM inhibited the expression of cytokines in the hippocampus induced by LPS.** **(A-D)** Mice were microinjected with the circControl/circDYM-GFP lentivirus in the hippocampus. One week after microinjection, mice were treated with LPS (1 mg/kg) or saline intraperitoneally injected for 5 successive days. The tissues of hippocampus was rinsed and homogenized in PBS. After centrifugation, the supernatant fluids were collected and assayed by ELISA assay for production of IL-6 **(A)**, IL-1β **(B)**, MCP-1 **(C)** and TNF-α **(D)**. All data were indicated as mean ± SEM. n=6 mice/group. (IL-6, circDYM: F_(1,20)_=10.531, P<0.01; LPS: F_(1,20)_=86.499, P<0.001; interaction: F_(1,20)_=11.166, P<0.01. IL-1β, circDYM: F_(1,20)_=34.667, P<0.001; LPS: F_(1,20)_=94.859, P<0.001; interaction: F_(1,20)_=42.490, P<0.001. MCP-1, circDYM: F_(1,20)_=19.588, P<0.001; LPS: F_(1,20)_=440.641, P<0.001; interaction: F_(1,20)_=8.497, P<0.01. TNF-α, circDYM: F_(1,20)_=38.866, P<0.001; LPS: F_(1,20)_=210.660, P<0.001; interaction: F_(1,20)_=21.697, P<0.001. ***p<0.001 versus circControl Control group; ^##^p<0.01 and ^###^p<0.001 versus circControl treated with LPS group).

**
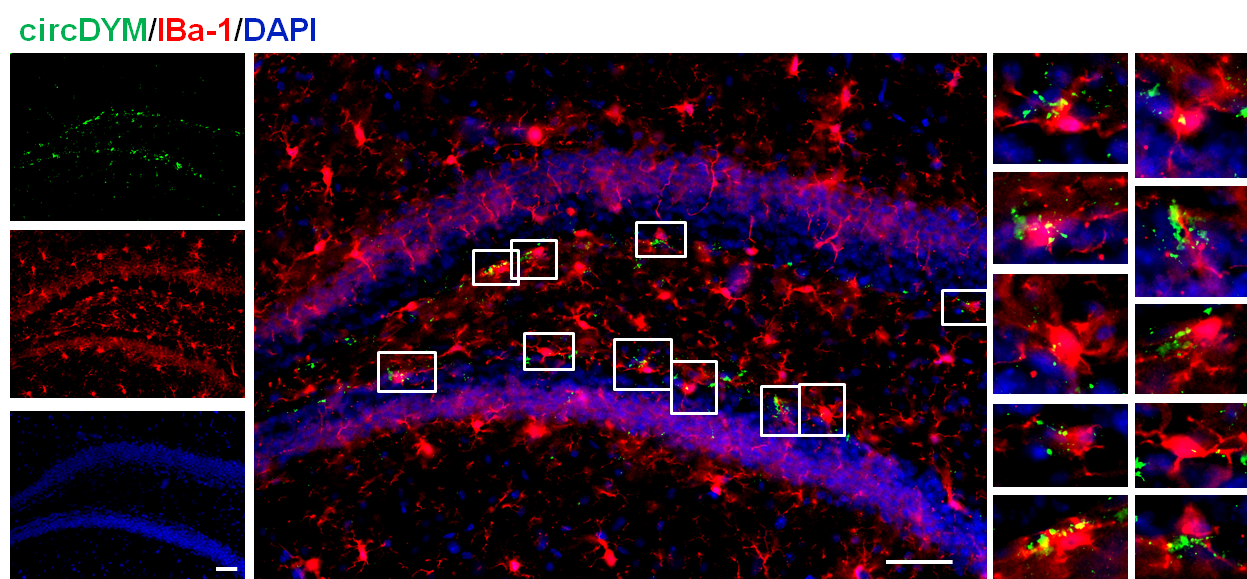
**

**Supplementary Figure 7. Co-localization of circDYM and Iba-1 in the hippocampus by immunostaining and FISH analysis.** Green, circDYM; Red, IBa-1; Blue, DAPI. Scale bar, 50 μm.

**
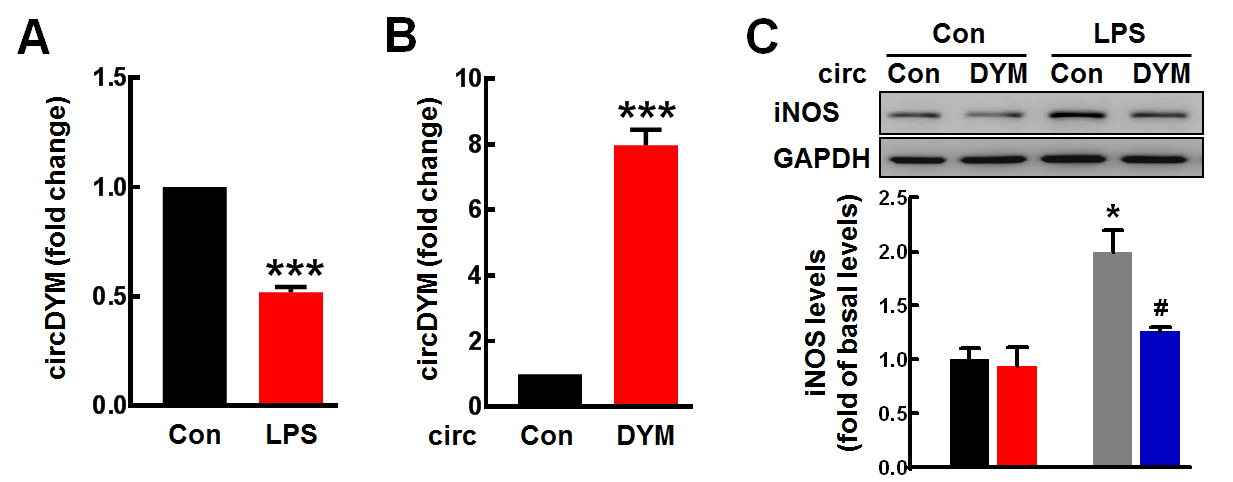
**

**Supplementary Figure 8. CircDYM expression inhibited microglial activation induced by LPS in BV-2 cells. (A)** Effect of LPS on the expression of circDYM in BV-2 cells. **(B)** Expression of circDYM in BV-2 cells transduced with the circDYM-GFP lentivirus. All data were presented as mean ± SEM of 3 independent experiments. ***p<0.001 versus Control using Student’s t-test. **(C)** Transduction with the circDYM-GFP lentivirus attenuated the iNOS expression induced by LPS in BV-2 cells. Cells were transduced with circControl or circDYM-GFP lentivirus for 24 h and then treated with LPS (100 ng/ml) for another 24 h. All data were presented as mean ± SEM of 3 independent experiments. (circDYM: F_(1,8)_=7.579, P<0.05; LPS: F_(1,8)_=20.661, P<0.01; interaction: F_(1,8)_=5.389, P<0.05. *p<0.05 versus circControl Control group; ^#^p<0.05 versus circControl treated with LPS group).

**
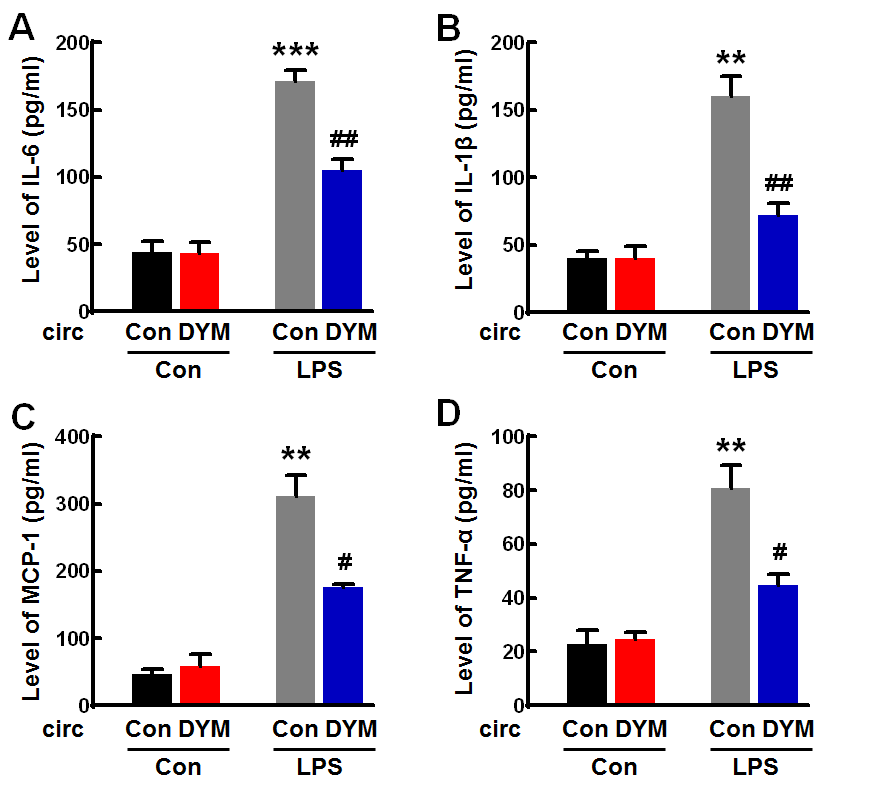
**

**Supplementary Figure 9. Transduction of cells with circDYM lentivirus inhibited the expression of cytokines induced by LPS in primary mouse microglial cells. (A-D)** Primary mouse microglial cells were transduced with circControl/circDYM-GFP lentivirus for 24 h and then were treated with LPS (100 ng/ml) for another 24 h. Cell culture supernatant were collected and assayed by ELISA assay for production of IL-6 **(A)**, IL-1β **(B)**, MCP-1 **(C)** and TNF-α **(D)**. All data were presented as mean ± SEM of 3 independent experiments. (IL-6, circDYM: F_(1,8)_=16.649, P<0.01; LPS: F_(1,8)_=131.742, P<0.001; interaction: F_(1,8)_=15.935, P<0.01. IL-1β, circDYM: F_(1,8)_=19.988, P<0.01; LPS: F_(1,8)_=58.673, P<0.001; interaction: F_(1,8)_=19.553, P<0.01. MCP-1, circDYM: F_(1,8)_=10.921, P<0.05; LPS: F_(1,8)_=106.610, P<0.001; interaction: F_(1,8)_=15.968, P<0.01. TNF-α, circDYM: F_(1,8)_=9.397, P<0.05; LPS: F_(1,8)_=49.310, P<0.001; interaction: F_(1,8)_=11.601, P<0.01. **p<0.01 and ***p<0.001 versus circControl Control group; ^#^p<0.05 and ^##^p<0.01 versus circControl treated with LPS group).


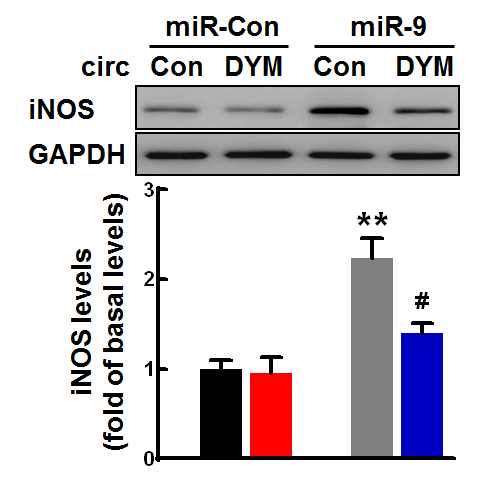


**Supplementary Figure 10. CircDYM expression inhibited microglial activation by targeting miR-9 in BV-2 cells.** Transduction with the circDYM-GFP lentivirus significantly inhibited the iNOS expression induced by miR-9 in BV-2 cells. All data were presented as mean ± SEM of 3 independent experiments. (circDYM: F_(1,8)_=7.483, P<0.05 miR-9: F_(1,8)_=27.356, p<0.01; interaction: F_(1,8)_=5.992, P<0.05. **p<0.01 versus miR-Control transduced with circControl group; ^#^p<0.01 versus miR-9 transduced with circControl group).


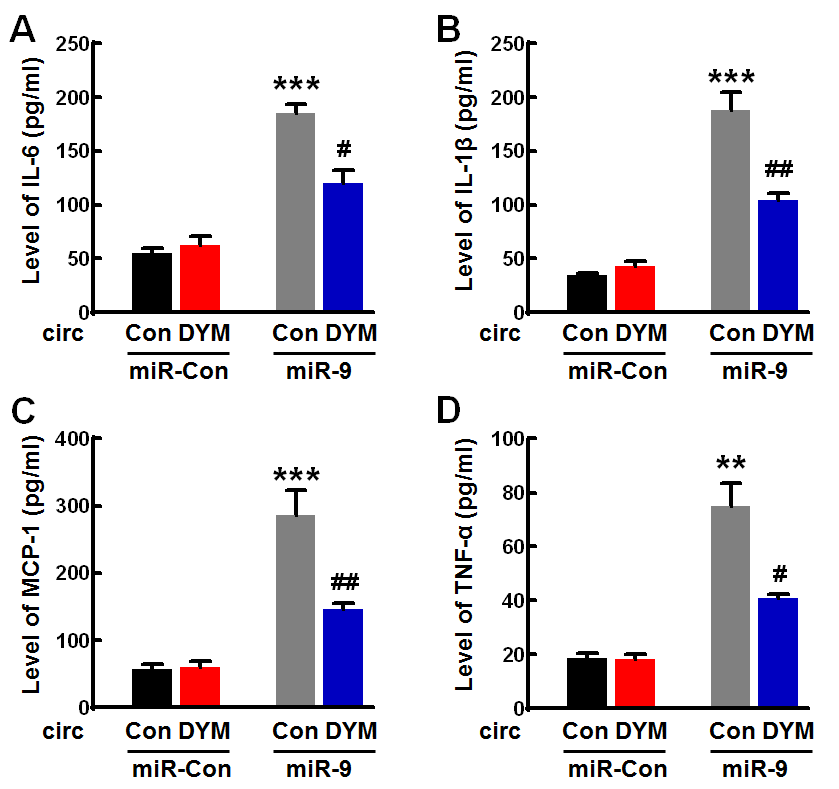


**Supplementary Figure 11. Transduction of cells with circDYM inhibited the expression of cytokines in primary mouse microglial cells transfected with miR-9. (A-D)** The primary mouse microglial cells were transfected with mimic miR-Control/miR-9 for 24 h and transduced with circControl/circDYM-GFP lentivirus for another 24 h. Cell culture supernatant ﬂuids were collected and assayed by ELISA assay for production of cytokines IL-6 **(A)**, IL-1β **(B)**, MCP-1 **(C)** and TNF-α **(D)**. All data were presented as mean ± SEM of 3 independent experiments. (IL-6, circDYM: F_(1,8)_=11.174, P<0.05; miR-9: F_(1,8)_=115.042, P<0.001; interaction: F_(1,8)_=17.139, P<0.01. IL-1β, circDYM: F_(1,8)_=31.894, P<0.001; miR-9: F_(1,8)_=221.831, P<0.001; interaction: F_(1,8)_=46.522, P<0.001. MCP-1, circDYM: F_(1,8)_=39.917, P<0.001; miR-9: F_(1,8)_=187.499, P<0.001; interaction: F_(1,8)_=42.643, P<0.001. TNF-α, circDYM: F_(1,8)_=14.171, P<0.01; miR-9: F_(1,8)_=74.169, P<0.001; interaction: F_(1,8)_=13.628, P<0.01. **p<0.01 and ***p<0.001 versus miR-Control transduced with circControl group; ^#^p<0.05 and ^##^p<0.01 versus miR-9 transduced with circControl group).


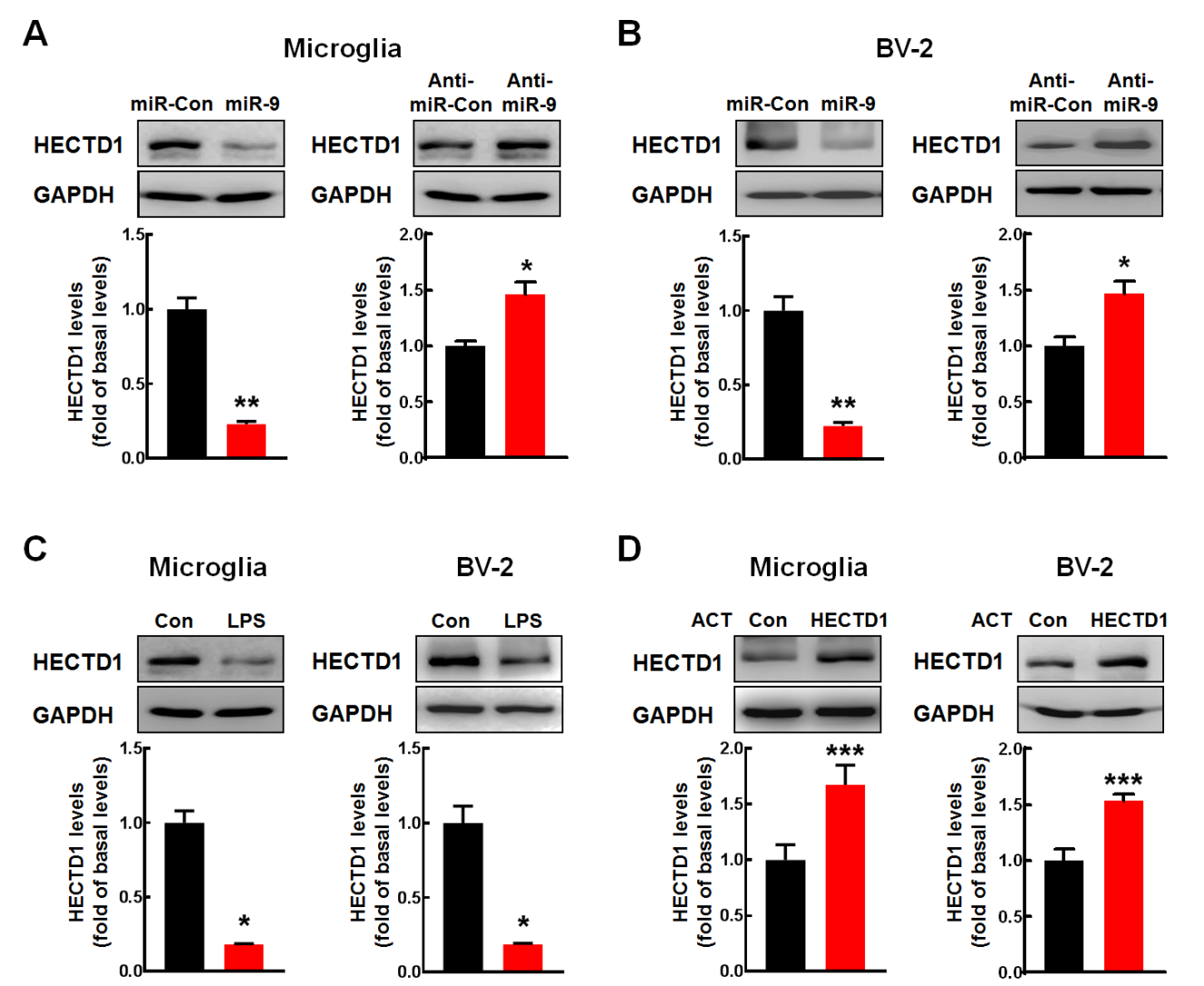


**Supplementary Figure 12. MiR-9 regulated microglial activation by targeting HECTD1. (A-B)** HECTD1 expression was evaluated in BV-2 and primary mouse microglial cells transfected with miR-Control/miR-9 or Anti-miR-Control/Anti-miR-9. All data were presented as mean ± SEM of 3 independent experiments. (*p<0.05 and **p<0.01 versus miR-Control or Anti-miR-Control using Student’s t-test). **(C)** Expression of HECTD1 induced by LPS in primary mouse microglial and BV-2 cells. Cells were treated with LPS (100 ng/ml) for 24 h. All data were presented as mean ± SEM of 3 independent experiments. *p<0.05 versus Control using Student’s t-test. **(D)** Expression of HECTD1 with Control-ACT/HECTD1-ACT detected by Western blot in primary mouse microglial and BV-2 cells. All data were presented as mean ± SEM of 3 independent experiments. ***p<0.001 versus Control-ACT using Student’s t-test.


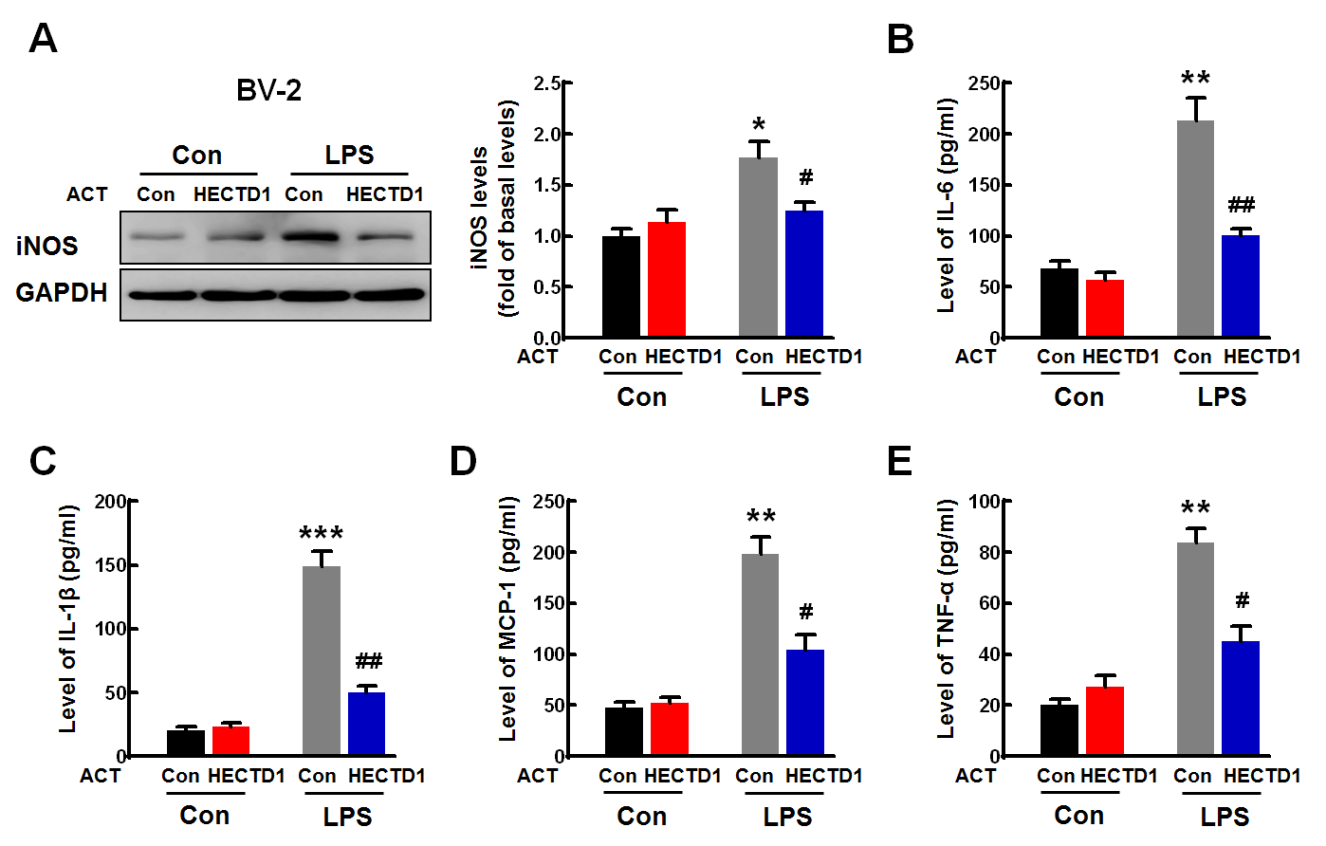


**Supplementary Figure 13. Overexpression of HECTD1 inhibited the expression of iNOS and cytokines in microglial cells induced by LPS.** **(A)** Overexpression of HECTD1 significantly inhibited the increased iNOS expression induced by LPS in BV-2 cells. All data were presented as mean ± SEM of 3 independent experiments. (HECTD1-ACT: F_(1,8)_=5.398, P<0.05; LPS: F_(1,8)_=21.784, P<0.01; interaction: F_(1,8)_=7.358, P<0.05). **(B-E)** Primary mouse microglial cells were transfected with HECTD1-ACT for 24 h and then were treated with LPS (100 ng/ml) for another 24 h. Cell culture supernatant ﬂuids were collected and assayed by ELISA assay for production of IL-6 **(B)**, IL-1β **(C)**, MCP-1 **(D)** and TNF-α **(E)**. All data were presented as mean ± SEM of 3 independent experiments. (IL-6, HECTD1-ACT: F_(1,8)_=23.731, P<0.01; LPS: F_(1,8)_=55.702, P<0.001; interaction: F_(1,8)_=15.931, P<0.01. IL-1β, HECTD1-ACT: F_(1,8)_=48.703, P<0.001; LPS: F_(1,8)_=126.800, P<0.001; interaction: F_(1,8)_=54.636, P<0.001. MCP-1, HECTD1-ACT: F_(1,8)_=13.266, P<0.01; LPS: F_(1,8)_=67.640, P<0.001; interaction: F_(1,8)_=16.117, P<0.01. TNF-α, HECTD1-ACT: F_(1,8)_=10.271, P<0.05; LPS: F_(1,8)_=67.870, P<0.001; interaction: F_(1,8)_=21.701, P<0.01). *p<0.05, **p<0.01 and ***p＜0.001 versus Control-ACT Control; ^#^p<0.05 and ^##^p<0.01 versus Control-ACT treated with LPS. Control-ACT: Control CRISPR Activation Plasmid (ACT); HECTD1-ACT: HECTD1 CRISPR Activation Plasmid (ACT).


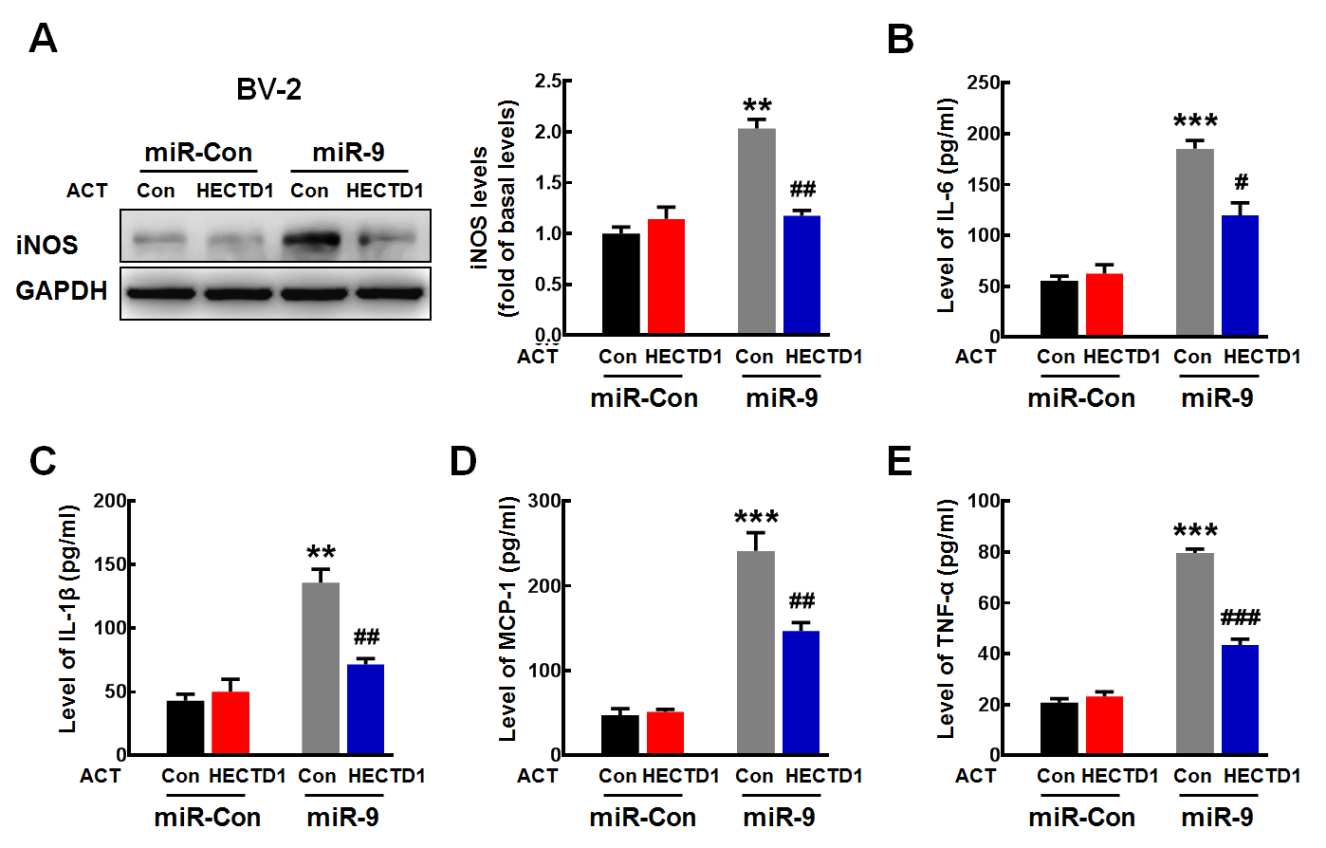


**Supplementary Figure 14. Overexpression of HECTD1 inhibited the expression of iNOS and cytokines in microglial cells transfected with miR-9. (A)** Overexpression of HECTD1 significantly inhibited the iNOS expression in BV-2 cells transfected with miR-9. All data were presented as mean ± SEM of 3 independent experiments. (HECTD1-ACT: F_(1,8)_=18.248, P<0.01; miR-9: F_(1,8)_=40.676, P<0.001; interaction: F_(1,8)_=36.182, P<0.001). **(B-E)** Primary mouse microglial cells were transfected with mimic miR-Control/miR-9 for 24 h and Control-ACT/HECTD1-ACT for another 24 h. Cell culture supernatant ﬂuids were collected and assayed by ELISA assay for production of cytokines IL-6 **(B)**, IL-1β **(C)**, MCP-1 **(D)** and TNF-α **(E)**. All data were presented as mean ± SEM of 3 independent experiments. (IL-6, HECTD1-ACT: F_(1,8)_=6.567, P<0.05; miR-9: F_(1,8)_=132.101, P<0.001; interaction: F_(1,8)_=5.444, P<0.05. IL-1β, HECTD1-ACT: F_(1,8)_=13.031, P<0.01; miR-9: F_(1,8)_=52.093, P<0.001; interaction: F_(1,8)_=20.203, P<0.01. MCP-1, HECTD1-ACT: F_(1,8)_=85.940, P<0.001; miR-9: F_(1,8)_=593.867, P<0.001; interaction: F_(1,8)_=77.852, P<0.001. TNF-α, HECTD1-ACT: F_(1,8)_=85.164, P<0.001; miR-9: F_(1,8)_=464.990, P<0.001; interaction: F_(1,8)_=111.097, P<0.001). **p<0.01 and ***p＜0.001 versus miR-Control transfected with Control-ACT group; ^#^p<0.05, ^##^p<0.01 and ^###^p<0.001 versus miR-Control transfected with Control-ACT group. Control-ACT: Control CRISPR Activation Plasmid (ACT); HECTD1-ACT: HECTD1 CRISPR Activation Plasmid (ACT).


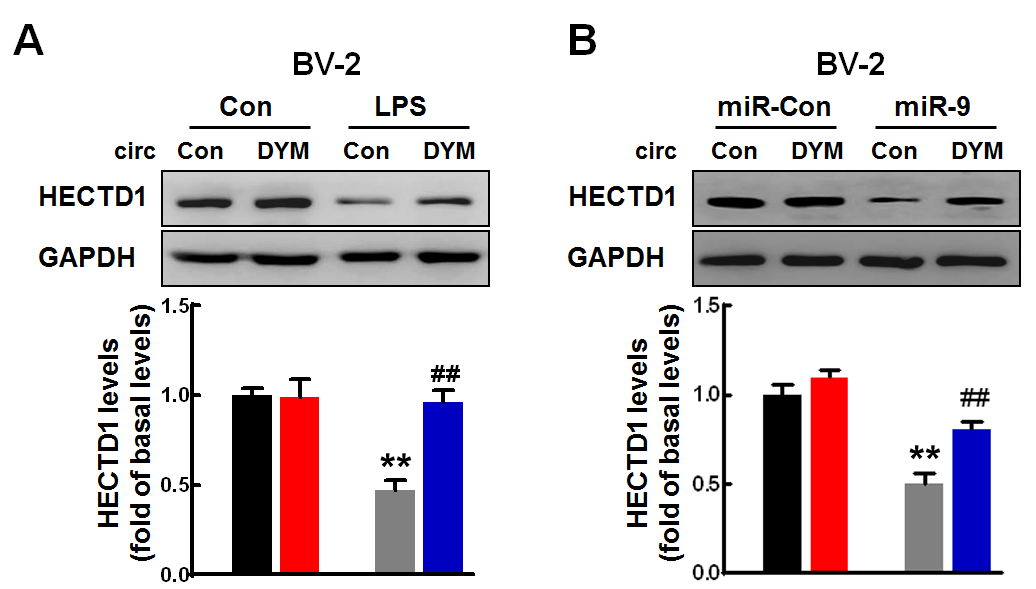


**Supplementary Figure 15. Effect of circDYM on the expression of HECTD1. (A)** Effect of circDYM overexpression on HECTD1 levels induced by LPS. Cells were transduced with the circControl/circDYM-GFP lentivirus for 24 h and treated with LPS (100 ng/ml) for another 24 h in BV-2 cells. All data were presented as mean ± SEM of 3 independent experiments. (circDYM: F_(1,8)_=11.843, P<0.01; LPS: F_(1,8)_=15.771, P<0.01; interaction: F_(1,8)_=12.934, P<0.01. **p<0.01 versus circControl Control group; ^##^p<0.01 versus circControl treated with LPS group). **(B)** circDYM overexpression significantly upregulated the HECTD1 expression in BV-2 cells transfected with miR-9. All data were presented as mean ± SEM of 3 independent experiments. (circDYM: F_(1,8)_=17.735, P<0.01; miR-9: F_(1,8)_=69.998, P<0.001; interaction: F_(1,8)_=5.493, P<0.05. **p<0.01 versus miR-Control transduced with circControl group; ^##^p<0.01 versus miR-9 transduced with circControl group).


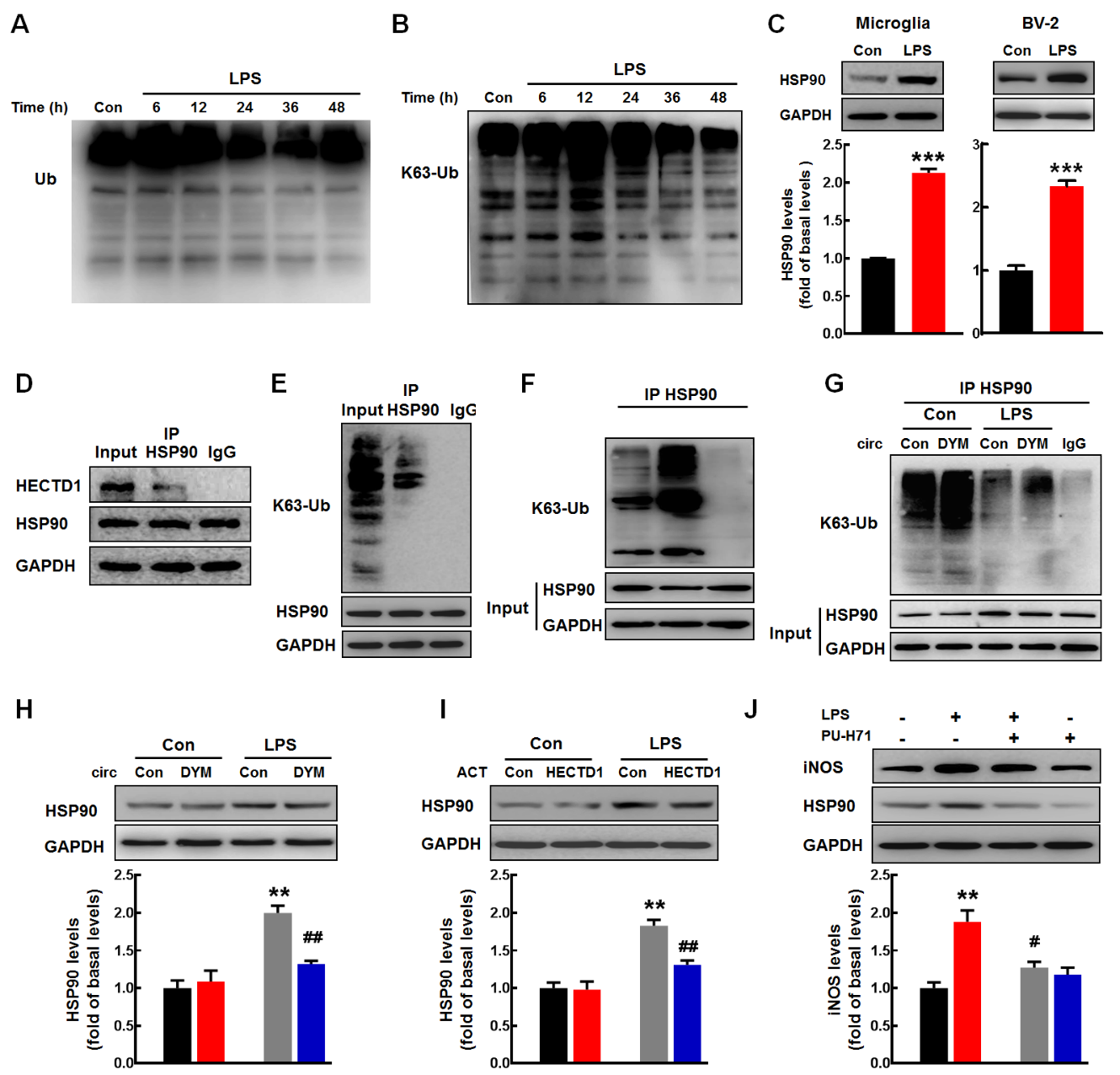


**Supplementary Figure 16. CircDYM/miR-9/HECTD1 regulated microglial activation via HSP90 ubiquitination *in vitro*.** **(A-B)** The ubiquitination **(A)** and K63 ubiquitination **(B)** induced by LPS in BV-2 cells. Cells were treated with LPS (100 ng/ml) for 24 h. **(C)** Expression of HSP90 induced by LPS in primary mouse microglial and BV-2 cells. All data were presented as mean ± SEM of 3 independent experiments. ***p<0.001 versus Control using student’s t-test. **(D)** Immunoblot analysis in the interaction between HECTD1 and HSP90 in BV-2 cells. **(E)** Immunoblot analysis in the interaction between HSP90 and K63 ubiquitination in BV-2 cells. **(F)** Overexpression of HECTD1 significantly increased the ubiquitination of HSP90 in BV-2 cells. **(G)** Overexpression of circDYM significantly upregulated the HSP90-binding K63 ubiquitination induced by LPS in BV-2 cells. **(H)** Overexpression of circDYM attenuated the increased HSP90 expression induced by LPS in BV-2 cells. Cells were transduced with circControl/circDYM-GFP lentivirus for 24 h and then were treated with LPS (100 ng/ml) for another 24 h. All data were presented as mean ± SEM of 3 independent experiments. (circDYM: F_(1,8)_=8.139, P<0.05; LPS: F_(1,8)_=35.399, P<0.001; interaction: F_(1,8)_=13.595, P<0.01. **p<0.01 versus circControl Control group; ^##^p<0.01 versus circControl treated with LPS group). **(I)** Overexpression of HECTD1 attenuated the HSP90 expression in BV-2 cells induced by LPS. Cells were transfected with Control-ACT or HECTD1-ACT for 24 h and were treated with LPS (100 ng/ml) for another 24 h. All data were presented as mean ± SEM of 3 independent experiments. (HECTD1-ACT: F_(1,8)_=11.219, P<0.05; LPS: F_(1,8)_=51.458, P<0.001; interaction: F_(1,8)_=9.679, P<0.05. **p<0.01 versus Control-ACT Control group; ^##^p<0.01 versus Control-ACT treated with LPS group). **(J)** HSP90 inhibitor PU-H71 significantly inhibited iNOS expression induced by LPS in primary mouse microglial cells. All data were presented as mean ± SEM of 3 independent experiments. (LPS: F_(1,8)_=29.517, P<0.01; PU-H71: F_(1,8)_=6.154, P<0.05; interaction: F_(1,8)_=19.517, P<0.01. **p<0.01 versus Control without PU-H71 group; ^#^p<0.05 versus LPS without PU-H71 group). Control-ACT: Control CRISPR Activation Plasmid (ACT); HECTD1-ACT: HECTD1 CRISPR Activation Plasmid (ACT).


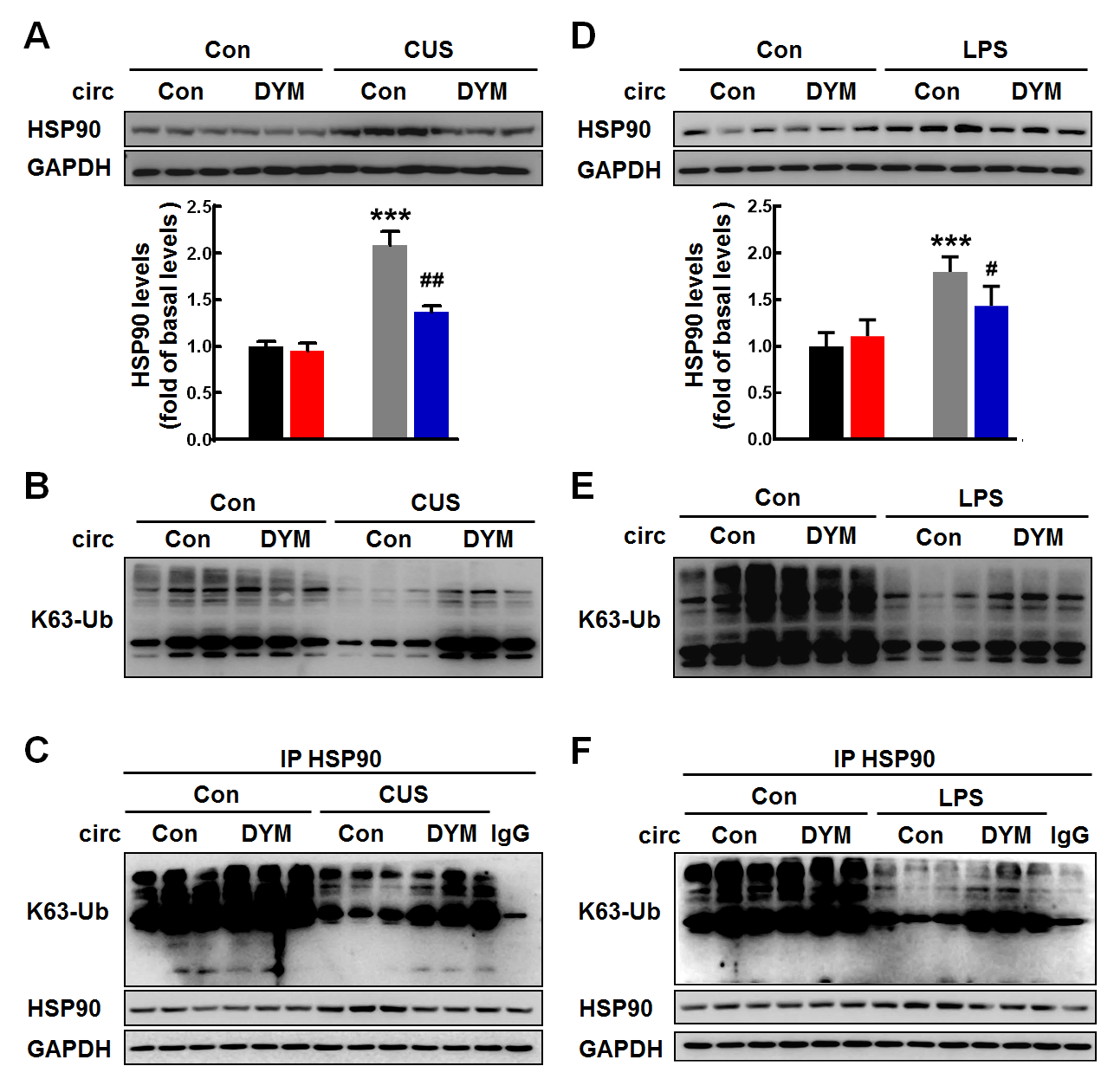


**Supplementary Figure 17. CircDYM regulated microglial activation via HSP90 ubiquitination *in vivo*.** **(A)** Overexpression of circDYM attenuated the HSP90 expression induced by CUS. Mice were microinjected with the circControl/circDYM-GFP lentivirus in the hippocampus. One week after microinjection, mice were exposed to CUS for 5 weeks. Three representative immunoblots were presented from 6 mice/group. All data were presented as mean ± SEM. (circDYM: F_(1,8)_=15.502, P<0.01; CUS: F_(1,8)_=60.291, P<0.001; interaction: F_(1,8)_=11.677, P<0.01. ***p<0.001 versus circControl Control group; ^##^p<0.01 versus circControl induced by CUS group). **(B)** Overexpression of circDYM significantly inhibited the K63 ubiquitination induced by CUS. **(C)** Overexpression of circDYM significantly inhibited the decreased HSP90 ubiquitination induced by CUS. **(D)** Overexpression of circDYM attenuated the HSP90 expression induced by LPS. Mice were microinjected with the circControl/circDYM-GFP lentivirus in the hippocampus. One week after microinjection, mice were treated with LPS (1 mg/kg) intraperitoneally injected for 5 successive days. Three representative immunoblots were presented from 6 mice/group. All data were presented as mean ± SEM. (circDYM: F_(1,8)_=4.588, P<0.05; LPS: F_(1,8)_=62.909 P<0.001; interaction: F_(1,8)_=9.403, P<0.01. ***p<0.001 versus circControl Control group; ^#^p<0.05 versus circControl induced by LPS group). **(E)** Overexpression of circDYM significantly inhibited the K63 ubiquitination induced by LPS. **(F)** Overexpression of circDYM significantly inhibited the decreased HSP90 ubiquitination induced by LPS.


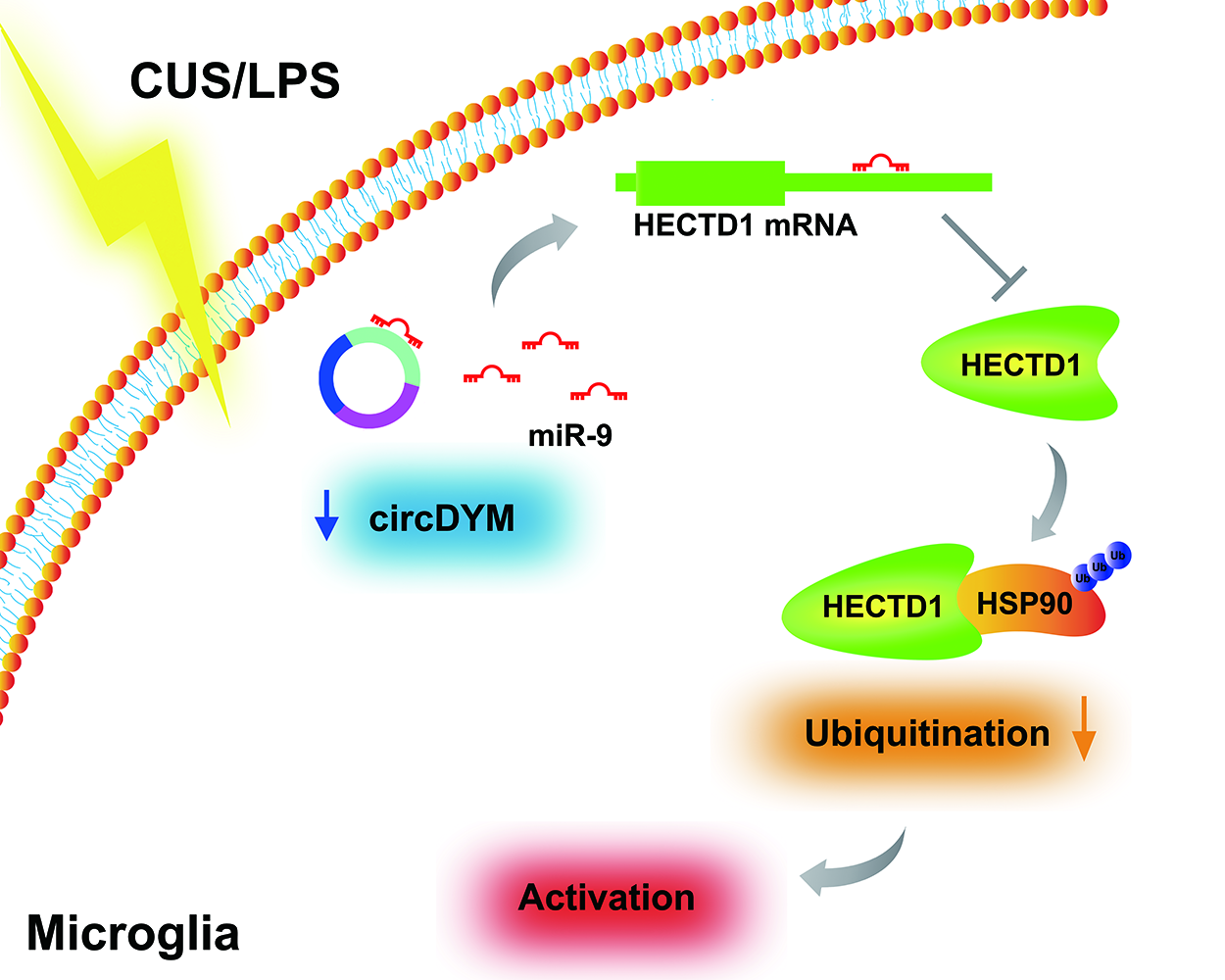


**Supplementary Figure 18. CircDYM/miR-9/HECTD1 regulated microglial activation via HSP90 ubiquitination induced by CUS or LPS.** CUS or LPS treatment decreased the expression of circDYM. Decreased expression of circDYM released miR-9 with concomitant downstream downregulation of HECTD1, resulting in the inhibition of HSP90 ubiquitination and the increase of microglial activation

**Supplementary Tables**

**Supplementary Table 1. The information of PCR primers.**

| **List of oligonucleotide sequences** | **5**' **> 3**' |
| --- | --- |
| GAPDH(human)-F | ACCATCTTCCAGGAGCGAGAT |
| GAPDH(human)-R | GGGCAGAGATGATGACCCTTT |
| GAPDH(mouse)-F | AGGTCGGTGTGAACGGATTTG |
| GAPDH(mouse)-R | TGTAGACCATGTAGTTGAGGTCA |
| circDYM(human)-F (divergent) | TGGAAGAATTGCTGTGCTGTT |
| circDYM(human)-R (divergent) | TGCACCAAGATTTCCTGTTCG |
| circDYM(mouse)-F (divergent) | GAAGAAAAGTCCCCCGGCAG |
| circDYM(mouse)-R (divergent) | AAGACCTTAGTTAGCGCAGCA |
| GAPDH(mouse-d)-F (divergent) | TGACCTCAACTACATGGTCTACA |
| GAPDH(mouse-d)-R (divergent) | CAAATCCGTTCACACCGACCT |
| circDYM(mouse-c)-F (convergent) | TGCTGCGCTAACTAAGGTCTT |
| circDYM(mouse-c)-R (convergent) | CTGCCGGGGGACTTTTCTTC |
| HECTD1(human)-F | ACGGTTGTACGCAAGGTTGA |
| HECTD1(human)-R | GGCGCTCTCTCATGATCTCC |
| HECTD1(mouse)-F | TTGAAACATGTCCACCTCGT |
| HECTD1(mouse)-R | CACGGGCTGTCACCTCTAAG |

**Supplementary Table 2. The information of Antibodies**

| **Antibody** | **Vendor** | **Catalog Number** |
| --- | --- | --- |
| Iba-1 | Wako | 019-19741 |
| HECTD1 | Proteintech, | 20605-1-AP |
| iNOS | Proteintech | 18985-1-AP |
| HSP90 | Proteintech | [13171-1-AP](http://www.ptgcn.com/products/HSP90-Antibody-13171-1-AP.htm) |
| Ub63 | abways | CY6579 |
| GAPDH | Proteintech, | 60004-1-Ig |
| Biotinylated goat-anti-rabbit IgG | Vector Laboratories | BA-1000 |
| Anti-digoxigenin antibody | Roche | 11207733910 |
| FITC-Streptavidin | Invitrogen | 434311 |
| anti-mouse IgG secondary antibody | Cell Signaling | 7076P2 |
| anti-rabbit IgG secondary antibody | Cell Signaling | 7074P2 |

**Supplementary Table 3. Participants’ demographics and clinical**

**characteristics**

|  | Normal (N=30) | Depression (N=50) | |  |
| --- | --- | --- | --- | --- |
|  | Mean±SEM | Mean±SEM | p | |
| Age (years) | 37.43±2.12 | 41.34±1.57 | 0.138 | |
| Gender (Male) (%, N) | 33.33% (10) | 40.0% (20) | 0.551 | |
| Family history | 1/30 | 13/50 | 0.011 | |
| First episode (%, N) | NA | 32.0% (16) |  | |
| On-set age (years) | NA | 33.93±1.59 |  | |
| Disease duration (months) | NA | 85.60±12.73 |  | |
| Current episode duration (months) | NA | 5.24±0.82 |  | |

Abbreviations: NA, Not Available

**Supplementary Table 4. Participants’ depression symptom characteristics**

|  | Normal (N=30) | Depression (N=50) | |  |
| --- | --- | --- | --- | --- |
|  | Mean±SEM | Mean±SEM | | p |
| HAMD-17 | 1.60±0.47 | 20.28±1.03 | 0.000 | |
| HAMD-Retardation | 0.33±0.15 | 6.44±0.41 | 0.000 | |
| HAMD-Suicide | 0 | 1.00±0.16 | 0.000 | |
| HAMD-Anxiety | 0.70±0.16 | 5.08±0.36 | 0.000 | |
| HAMD-Sleep | 0.30±0.20 | 3.94±0.35 | 0.000 | |
| HAMD-Weight | 0 | 0.44±0.11 | 0.000 | |
| TEPS | 65.17±2.07 | 56.68±1.69 | 0.002 | |
| TEPS-A | 35.40±1.08 | 30.80±1.00 | 0.004 | |
| TEPS-C | 29.77±1.16 | 25.88±0.88 | 0.009 | |
| PAS | 15.30±1.28 | 28.52±1.28 | 0.000 | |
| SAS | 10.10±0.86 | 15.60±1.00 | 0.000 | |
| HAMA | 2.03±0.67 | 16.74±1.04 | 0.000 | |

# Abbreviations: HAMD-17, 17-item Hamilton Depression Scale; HAMD-Retardation, Hamilton Depression Scale-Retardation factor; HAMD-Suicide, Hamilton Depression Scale-Suicide factor; HAMD-Anxiety, Hamilton Depression Scale-Anxiety/somatization factor; HAMD-Sleep, Hamilton Depression Scale-Sleep disorder factor; HAMD-Weight, Hamilton Depression Scale- Loss of weight factor; TEPS, Temporal Experience of Pleasure Scale; TEPS-A, Temporal Experience of Pleasure Scale-Anticipatory Pleasure; TEPS-C, Temporal Experience of Pleasure Scale-Consummatory Pleasure; PAS, Physical Anhedonia Scale; SAS, Social Anhedonia Scale; HAMA, Hamilton Anxiety Scale.

**Supplementary Table 5. Participants’ social-psychological characteristics**

|  | Normal (N=30) | | Depression (N=50) | |  |
| --- | --- | --- | --- | --- | --- |
|  | Mean±SEM | | Mean±SEM | | p |
| RFQ-promote | | 19.43±0.64 | 16.92±0.51 | 0.003 | |
| CTQ | | 34.70±1.58 | 43.08±1.68 | 0.001 | |
| CTQ-PA | | 5.67±0.23 | 6.54±0.35 | 0.039 | |
| CTQ-EA | | 6.83±0.40 | 8.14±0.49 | 0.044 | |
| CTQ-SA | | 5.47±0.18 | 6.36±0.34 | 0.022 | |
| CTQ-PN | | 7.70±0.56 | 9.72±0.46 | 0.007 | |
| CTQ-EN | | 9.00±0.77 | 12.44±0.74 | 0.002 | |

Abbreviations: RFQ, Regulatory Focus Questionnaire; CTQ, Childhood Trauma Questionnaire; CTQ-PA, Childhood Trauma Questionnaire-Physical Abuse; CTQ-EA, Childhood Trauma Questionnaire-Emotional Abuse; CTQ-SA, Childhood Trauma Questionnaire-Sexual Abuse; CTQ-PN, Childhood Trauma Questionnaire-Physical Neglect; CTQ-EN, Childhood Trauma Questionnaire-Emotional Neglect.
